# Towards a Quantitative Mechanistic Understanding of Localized Pulmonary Tissue Retention—A Combined In Vivo/In Silico Approach Based on Four Model Drugs

**DOI:** 10.3390/pharmaceutics12050408

**Published:** 2020-04-29

**Authors:** Anneke Himstedt, Clemens Braun, Sebastian Georg Wicha, Jens Markus Borghardt

**Affiliations:** 1 Department of Clinical Pharmacy, Institute of Pharmacy, University of Hamburg, 20146 Hamburg, Germany; anneke.himstedt@uni-hamburg.de; 2 Drug Discovery Sciences, Boehringer Ingelheim Pharma GmbH & Co. KG, 88397 Biberach, Germany; clemens.braun@boehringer-ingelheim.com

**Keywords:** lung retention, pharmacokinetics, pulmonary blood flow, tissue affinity, semi-mechanistic PK modelling, trachea, bronchi, alveolar, lung concentration

## Abstract

Increasing affinity to lung tissue is an important strategy to achieve pulmonary retention and to prolong the duration of effect in the lung. As the lung is a very heterogeneous organ, differences in structure and blood flow may influence local pulmonary disposition. Here, a novel lung preparation technique was employed to investigate regional lung distribution of four drugs (salmeterol, fluticasone propionate, linezolid, and indomethacin) after intravenous administration in rats. A semi-mechanistic model was used to describe the observed drug concentrations in the trachea, bronchi, and the alveolar parenchyma based on tissue specific affinities (K_p_) and blood flows. The model-based analysis was able to explain the pulmonary pharmacokinetics (PK) of the two neutral and one basic model drugs, suggesting up to six-fold differences in K_p_ between trachea and alveolar parenchyma for salmeterol. Applying the same principles, it was not possible to predict the pulmonary PK of indomethacin, indicating that acidic drugs might show different pulmonary PK characteristics. The separate estimates for local K_p_, tracheal and bronchial blood flow were reported for the first time. This work highlights the importance of lung physiology- and drug-specific parameters for regional pulmonary tissue retention. Its understanding is key to optimize inhaled drugs for lung diseases.

## 1. Introduction

Pulmonary drug delivery is the preferred administration route for treatment of respiratory disorders like asthma or chronic obstructive pulmonary disease. The reason is that oral drug inhalation can provide pulmonary selectivity for locally acting drugs and long-lasting pulmonary efficacy, as can be seen for long-acting β_2_-receptor agonists (LABAs) and long-acting muscarinic receptor agonists (LAMAs). To achieve long-lasting pulmonary efficacy, there are mainly two strategies applied to maintain pulmonary exposure over a long period of time, namely slow dissolution (e.g., inhaled fluticasone propionate [1]) or retention by high tissue affinity (postulated e.g., for the LABA salmeterol [2]). However, a recent publication suggested that retention due to high lung tissue affinity is preferable over slow dissolution in terms of target exposure [3]. One reason is that slow dissolution may lead to noticeable drug loss in the conducting airways via the mucociliary clearance.

The lung is a very heterogeneous organ with various structural differences between the conducting airways and the alveolar region [4]. In addition, the alveolar region is stronger perfused compared to the conducting airways, as the alveolar region is perfused by the pulmonary circulation, whereas the conducting airways are perfused by the systemic circulation. All these physiological differences potentially influence drug pharmacokinetics (PK), raising the question if total lung concentrations are a valid surrogate for target-site concentrations. For example, β_2_-receptors are expressed in all lung regions [5], yet the relaxation of smooth muscle cells by inhaled sympathomimetics is driven by receptor activation in the conducting airways. This means that only the drug concentrations in the conducting airways elicit the desired effect. Therefore, it is key to understand the PK in the local tissues and not only for the complete lung.

The local pulmonary tissue PK and therefore the local tissue retention is determined by two aspects, first the tissue affinity and second the local perfusion. Due to experimental difficulties, investigations of pulmonary tissue retention have been mostly qualitative in nature [6,7] or were based on empirical estimation of tissue distribution or absorption rate constants [8,9]. A more mechanistic quantitative determination of pulmonary disposition kinetics remains challenging for various reasons: First, after inhalation the variability in the PK is typically much higher compared to other routes of administration, so that a larger data set is required to infer on the pulmonary tissue retention. Second, after inhalation or intratracheal administration, there are many interacting PK processes, such as pulmonary deposition or the mucociliary clearance. All these processes confound the identification of the characteristics of single processes—here the pulmonary tissue retention [4]. Furthermore, low solubility drugs provide the additional challenge that there is no convenient way of differentiating between undissolved and dissolved drug in the lung. All of this makes it challenging to quantitatively determine the extent of the pulmonary tissue retention. By switching to intravenous (i.v.) administration to study distribution into different lung tissues one avoids most of these challenges, i.e., reduces the variability in the PK and removes confounding pulmonary PK processes.

The objective of this work was to better understand pulmonary retention in different lung regions by combining well-designed in vivo tissue distribution studies with a PK model-based analysis of plasma and tissue concentration measurements. Finally, the aim was to provide accurate estimates for tissue affinity and perfusion for different regions of the lung.

To achieve this, four drugs with varying physicochemical properties, namely salmeterol (SAL), fluticasone propionate (FP), linezolid (LIN), and indomethacin (IND), were intravenously administered to rats and the concentrations in plasma, trachea, bronchi and alveolar parenchyma were measured. These model drugs were chosen based on tolerability and relevance while covering a range of physicochemical properties (one basic, two neutral, and one acidic drug). Both SAL and FP are used to treat lung diseases, and LIN is often used for the treatment of pulmonary infections. As there are only few acidic drugs on the market, which are targeting pulmonary structures, IND was chosen based on tolerability and ease of acquisition. The data from these in vivo PK studies was further investigated regarding their disposition in the different lung tissues using a semi-mechanistic model accounting for both physiological and drug-specific properties. Since to date, no separate quantification of blood flows in trachea and bronchi has been reported in the literature, pulmonary blood flows were estimated based on the available data of all four drugs. Finally, based on the resulting understanding of the local concentration–time profiles, current plasma concentration–based practices for determining pharmacodynamic (PD) parameters were evaluated.

## 2. Materials and Methods

### 2.1. Chemicals

FP, SAL, LIN, and IND were sourced from the in-house compound dispensary at Boehringer Ingelheim Pharma GmbH & Co. KG, (Biberach, Germany). Deuterated internal standards were purchased for IND (indomethacin-d4, Biomol GmbH, Hamburg, Germany), FP (fluticasone propionate-d5, Santa Cruz Biotechnology Inc., Dallas, TX, USA), and LIN (linezolid-d3, Biomol GmbH). Structures and key properties of the drugs are given in the supplementary material (Supplementary material 1, Figure S1, Table S1).

### 2.2. Study Design

The in vivo PK studies were designed based on concentration–time profiles from exploratory i.v. cocktail PK studies (data not shown). Model-based analyses were performed to ensure that (a) the dose is sufficient to achieve concentrations above the lower limit of quantification in plasma and tissues over the whole study period, and (b) the timing of tissue sampling allows for accurate estimation of partition coefficients and localized pulmonary blood flows and therefore adequately capturing the shape of tissue concentration–time profiles. For the latter analysis, a stochastic simulation and estimation approach (SSE) was used to evaluate identifiability, bias and imprecision of the model parameters prior to performing the in vivo studies (Supplementary Material 1, Section 2, Tables S2 and S3, Figures S2 and S3). In the final studies, the drugs were administered via intravenous infusion to reach near-steady state conditions in the study period and minimize residual variability due to potential imprecision of the sampling time.

### 2.3. Animal Studies

Male Han Wistar rats, weighing 250 to 332 g, purchased from Janvier Labs (Le Genest-Saint-Isle, France) were used for the in vivo studies. All animal care and experimental procedures at Boehringer Ingelheim were conducted in compliance with the German and European Animal Welfare Act (EU Directive 2010/63/EU) and were approved by the Regierungspräsidium Tübingen as the responsible local German authority (reference number 14-009-G).

The infusion studies were performed in anesthetized animals. Briefly, the rats were anesthetized via whole body exposure to anesthetic gas (2–5% Isoflurane, 2.5 L/min Oxygen). Following anaesthetization, rats were intubated, placed in supine position on a heated device (39 °C), and the spontaneously breathing rats were connected to an anesthetic gas supply (1.5–2.5% Isoflurane, 2–2.5 L/min O2). Thereafter, rats received a subcutaneous bolus of metamizol (100 mg/kg). Body temperature was controlled. Placement of catheters for drug infusion and blood sampling was not started before a body temperature of at least 36.5 °C was reached. Unilaterally, the carotid artery and the jugular vein were prepared and catheters were placed. The carotid catheter was used for blood pressure monitoring to adjust anesthesia by changing isoflurane concentration as well as for collection of blood samples. The jugular catheter was used for constant infusion over one hour (infusion rate 10 mL/h/kg) using a standard infusion pump. Blood samples (volume 100 µL) were collected in EDTA-tubes at the assigned time points. Plasma samples were prepared and subsequently stored at −20 °C. At the end of the in-life part, rats were exsanguinated.

### 2.4. Tissue Preparation

**Figure 1:**
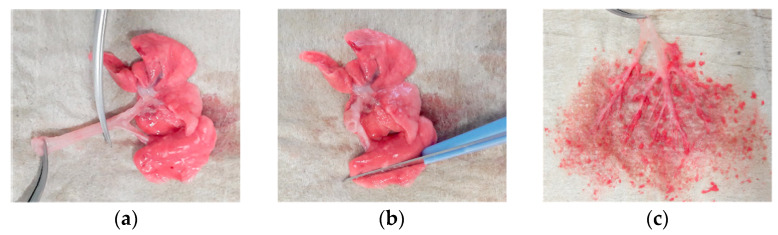
Preparation of tissue samples. (**a**) Sampling of the trachea from the intact lungs; (**b**) cutting a slice from the lateral part of the left lung; (**c**) bronchial sample after removal of the surrounding parenchyma.

Immediately after exsanguination, the lungs including trachea and larynx were removed en bloc, rinsed in saline, blotted dry, and weighed. The preparation of the lung was performed after placing the lung on weighed cellulose swabs to collect leaking fluid during preparation (Figure 1). The trachea including the larynx was cut just above the first airway bifurcation and transferred to a weighed vial. A small piece (30–60 mg) of parenchyma was cut with a scalpel from the lateral part of the left lung. For preparation of the bronchial sample, the remaining lung was held in place with forceps at the bifurcation while the parenchyma was squished by gently knocking with the back part of curved forceps. Afterwards, the destroyed parenchyma was carefully stripped from the bronchi up to the third airway generation. Finally, the remaining tissue and the cellulose swabs were collected for further analysis. Having finished the preparation, all collected samples were weighed, transferred to 7 mL Precellys^®^ tubes (Bertin Instruments, Montigny-le-Bretonneux, France), and 4 parts of acetonitrile/methanol (1:1) solution were added. Samples were homogenized using a Precellys^®^ homogenizer. After centrifugation, supernatants were stored at −20 °C.

### 2.5. Bioanalysis

Drug concentrations in plasma and tissue homogenates were determined by HPLC-MS/MS (reverse-phase HPLC coupled with a Thermo Scientific^TM^ TSQ Altis^TM^ triple quadrupole mass spectrometer (Thermo Fisher Scientific, Bremen, Germany) or a SCIEX QTRAP 6500 (AB Sciex, Darmstadt, Germany)). Prior to bioanalysis, plasma and tissue samples were spiked with internal standard solution and diluted with acetonitrile for protein precipitation. A more detailed description of analytical methods can be found in the supporting information (Supplementary Material 1, Section 5, Figures S4–S8).

### 2.6. Modelling and Simulation

**Figure 2:**
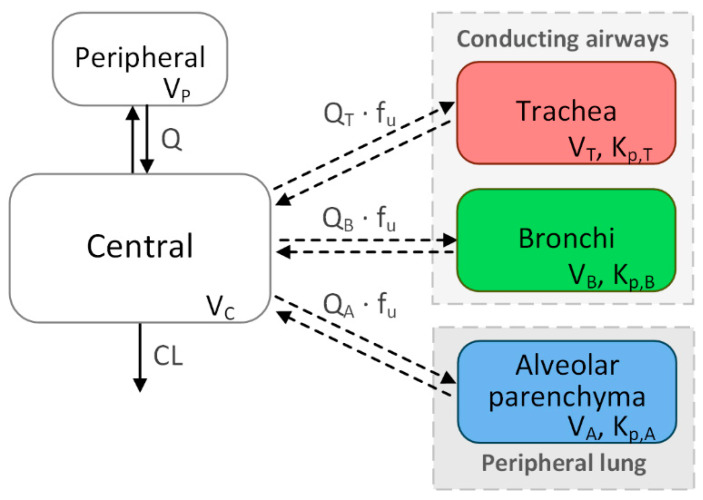
Structure of the pharmacokinetic (PK) model. CL: systemic clearance, V_C_: central volume of distribution (Vd), Q: intercompartmental clearance, V_P_: peripheral Vd; Q_T_, Q_B_, and Q_A_ represent the blood flow to the trachea, bronchi and alveolar parenchyma, respectively. f_u_: fraction unbound in plasma, V_T_: weight of the trachea, V_B_: weight of the bronchi, V_A_: weight of the alveolar parenchyma. K_p,T_, K_p,B_, and K_p,A_ denote the tissue-to-plasma partition coefficients for the respective tissues.

The model-based PK analysis was carried out in Phoenix WinNonlin^TM^ 7.0 (Certara, L.P., Princeton, NJ, USA). PK parameters of the semi-mechanistic model (Figure 2) were estimated in a two-stage approach. As a first step, the systemic PK parameters were estimated based on the plasma concentration–time data, resulting in empirical one- or two-compartment models depending on the drug characteristics. In the second step, the tissue-specific parameters were estimated on top of the fixed systemic parameters using the tissue concentration–time profiles resulting from the four time points of tissue sampling.

As the systemic disposition model already constitutes distribution into the lung, the pulmonary compartments were implemented as “virtual” compartments, i.e., these compartments did not contribute to the mass flow of the systemic compartments. In contrast to the systemic disposition model, the parameters involving the tissue compartments were chosen based on the underlying physiology [10].

The volume of distribution (Vd) for each of the pulmonary compartments was fixed to the weight of each tissue in proportion to the bodyweight. The according weight for the alveolar parenchyma equated to 0.004 kg/kg bodyweight, assuming that the parenchyma represents approximately 80% of total lung tissue [11] (0.5% of bodyweight, [12]). The remaining 20% (0.001 kg/kg bodyweight) were assumed to represent the conducting airways including bronchi and trachea. For the trachea, the implemented weight corresponded to the individual sample weights (Supplementary Material, Table S4) in relation to the bodyweight (mean value, 0.0002 kg/kg bodyweight), and the bronchi corresponded to the remaining 0.0008 kg/kg bodyweight.

The blood flows to the lung tissues were estimated. As the blood flow should be independent of the infused drug, these parameters were estimated simultaneously with the combined data from all drugs. Plausible initial values for the estimation were calculated based on literature data on cardiac output (alveolar parenchyma) and bronchial circulation (bronchi and trachea, calculated proportional to tissue weight) [12]. The blood flows were scaled with the fraction unbound in plasma, i.e., it was assumed that only unbound drug is permeating into tissue. Tissue affinity was described by tissue-to-plasma partition coefficients (K_p_), which were estimated separately for each of the three lung tissues. The model code is provided in the supporting information (Supplementary Material 2).

For comparison with common approaches that consider the lung as a single compartment [13,14,15,16,17], the same analysis was also carried out without separation of trachea, bronchi and alveolar parenchyma. To this end, full lung concentrations were calculated from the concentrations of all three lung tissues plus concentrations measured in the remaining lung tissue, weighted based on relative tissue size (Supplementary Material 1, Section 7), and subsequently used for estimation of lung-specific parameters. Model predictions of both variants were compared to the observed data using goodness-of-fit plots and the coefficients of determination.

Pulmonary absorption half-lives (t_½,pul_) for each lung region were calculated from the resulting model parameters:

t_½,pul_ = ln(2)/k_a,pul_, (1)

with k_a,pul_ being the absorption rate constant representing the unidirectional drug transfer from the lung tissue to the systemic circulation:

k_a,pul_ = (Q_pul_∙f_u,plasma_)/(V_pul_·K_p,pul_), (2)

Q_pul_ being the respective estimated pulmonary blood flow scaled with the fraction unbound in plasma (f_u,plasma_). V_pul_ denotes the Vd of the respective lung region, and K_p,pul_ is the respective estimated tissue-to-plasma partition coefficient. The ratio of f_u,plasma_/K_p,pul_ represents the free fraction in the respective pulmonary tissue.

Since rodent studies are typically performed to better predict the human situation and all four model drugs are designed for treatment of humans, allometric scaling to human was performed. This was done by the fixed exponent method, assuming an exponent of 0.75 for the blood flow:

Q_pul,human_ = Q_pul,rat_·SF·(BW_human_/BW_rat_)^0.75^, (3)

and 1 for the pulmonary volume of distribution:

V_pul,human_ = V_pul,ra_t·SF·(BW_human_/BW_rat_)^1^, (4)

with BW_rat_ (0.28 kg, mean bodyweight of animals used in the studies, Supplementary material, Table S4) and BW_human_ (70 kg) as bodyweights for rats and humans, respectively. SF denotes a scaling factor (BW_rat_/BW_human_) to account for the bodyweight normalization. K_p,pul_ was assumed to be conserved between species [18]. The respective extrapolated parameters were again used as input to Equation (2) to calculate the human absorption rate constant, which was used to calculate the human pulmonary absorption half-life according to Equation (1).

PK studies are often performed to infer on the expected efficacy by correlating the measured drug concentrations to the observed effect. As target-site concentrations in tissues are difficult to measure, plasma concentrations are typically used as a surrogate to quantify the concentration–response relationship. However, this approach does not consider potentially delayed concentration changes at the target site compared with plasma. To investigate the influence of this distributional delay, the developed PK model for SAL was further expanded by an E_max_ model, linking the predicted unbound concentration in the bronchi (C_u,bronchi_) to the effect, assuming the bronchi to be the target tissue for SAL [19]:

E [%] = (E_max_·C_u,bronchi_)/(EC_50,free_ + C_u,bronchi_). (5)

Here, E denotes the effect associated with a given C_u,bronchi_, E_max_ represents the maximum attainable effect (here 100%), and the EC_50,free_ represents the unbound concentration needed to achieve half-maximal effect. To exemplify the impact for the example of SAL, the EC_50_ for SAL was taken from Hendrickx et al. [8], who measured the inhibition of methacholine-induced bronchoconstriction and found an EC_50_ of 36 nM (total lung concentration). This value was scaled to unbound concentrations using the fraction unbound in plasma (f_u,plasma_) and the estimated K_p_ in bronchi (K_p,B_):

f_u,bronchi_ = f_u,plasma_/K_p,B_, (6)

assuming that unbound concentrations at equilibrium are the same in plasma and tissue.

Simulations were carried out to mimic the determination of unbound plasma EC_50_ (EC_50,free_) of SAL by dose escalation at four different time points (0.25 h, 0.75 h, 2 h, and 4 h). This was done by simulating unbound plasma and tissue concentration–time profiles, as well as the effect over time for a wide dose range. The simulated effect for each dose at the selected time point was then correlated to the corresponding unbound plasma concentration. The resulting concentration–response relationship was then used to determine the plasma EC_50,free_. For better illustration, the resulting parameter estimates were normalized to the true EC_50,free_. The determination of PD parameters was performed in R (version 3.3.2) [20].

## 3. Results

### 3.1. In Vivo PK Studies

Based on the results from the SSE analysis (Supplementary Material, Section 2), four time points were chosen for sampling of the lung tissues (three samples per time point and tissue), resulting in 12 samples per tissue and drug. The first samples were taken during the infusion (0.25 h and 0.75 h) to capture potential initial delayed increases in tissue concentrations. The two additional samples were taken at 2 h and 4 h after start of infusion, corresponding to 1 h and 3 h after the end of the infusion. Plasma was sampled until the end of the respective experiment, resulting in an average (range) of 67 (58–70) plasma samples per drug (see Supplementary Material, Table S4), which were mainly sampled within the first hour.

**Figure 3:**
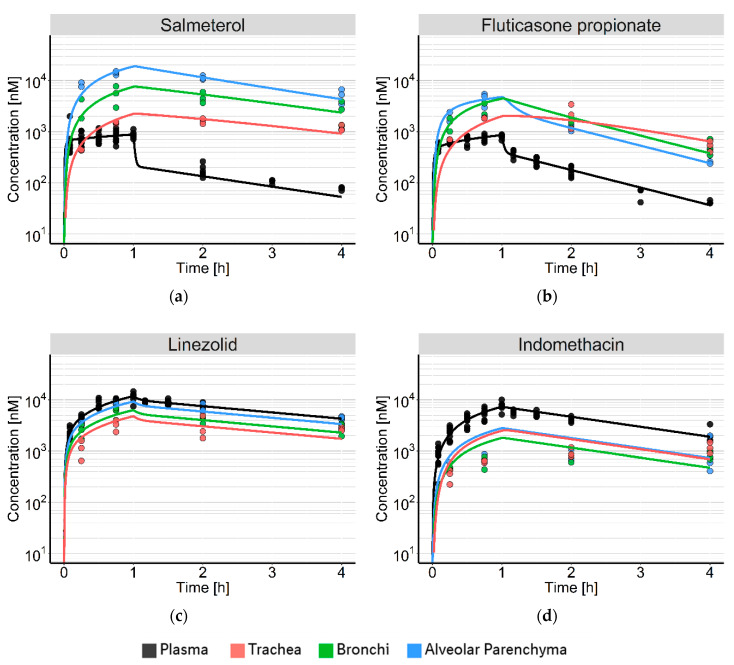
Concentration–time profiles of (**a**) salmeterol; (**b**) fluticasone propionate; (**c**) linezolid, and (**d**) indomethacin. Dots represent the observed concentrations; solid lines show the model-based prediction. Plasma concentrations are shown in black; concentrations in the trachea, bronchi, and alveolar parenchyma are shown in red, green and blue, respectively.

Plasma concentrations of SAL, FP, and LIN showed a bi-exponential decline after stopping the infusion, while IND showed mono-exponential decay (Figure 3). While pulmonary tissue concentrations of LIN were only slightly lower than plasma concentrations, for the acidic IND up to ten-fold lower tissue concentrations were measured. In contrast, tissue concentrations of both the neutral FP and the basic SAL were generally higher than the corresponding plasma concentration measurements.

IND concentrations were comparable in all pulmonary tissues. In contrast, the raw data of SAL, FP, and LIN indicated differences in magnitude and time-course of concentrations between trachea, upper bronchial tree, and alveolar parenchyma. This was most noticeable for SAL, which showed up to 20-fold higher concentrations in the alveolar parenchyma compared to the trachea. All drugs showed a distributional delay in the trachea.

### 3.2. Model-Based PK Analysis

The bi-exponential decline of plasma concentrations of SAL, FP and LIN was best described by an empirical two-compartment model. Since the plasma sampling of SAL shortly after stopping the infusion was not sufficient to support the estimation of all PK parameters of the systemic disposition model, additional data from another PK study was included in the analysis (Supplementary Material 1, Section 4). For IND, a one-compartment model was sufficient to capture the systemic PK.

In the second step, the tissue-specific parameters (K_p_ and tissue blood flows) were estimated on top of the fixed systemic disposition model. Estimates for K_p_ varied depending on both the investigated drug and tissue. In accordance with the raw data, K_p_ values for IND were comparable between all three lung tissues and estimated to range between 0.249–0.384, indicating lower tissue affinity compared to the affinity to plasma proteins. FP also showed similar affinity for all three pulmonary tissues (K_p_ between 5.21 and 6.64). While the K_p_ estimates for all tissues were higher for SAL compared to LIN, both drugs showed higher concentrations in the alveolar parenchyma compared to the conducting airways, with the lowest concentrations found in the trachea. SAL showed the strongest divergence in K_p_ values between tissues, with a six-fold higher affinity for the alveolar parenchyma than the trachea (39.3 vs. 6.52, respectively). Pulmonary blood flows could successfully be estimated (<22% CV) when data of all drugs was combined for simultaneous fitting. The tracheal blood flow was estimated to be 0.054 L/h/kg (14.3% CV), the bronchial blood flow amounted to 0.777 L/h/kg (21.5% CV). The parameter estimates for systemic and tissue PK can be found in Table 1.

The model-based analysis adequately explained the tissue-specific pulmonary disposition of the neutral and basic model drugs (SAL, FP, and LIN). However, applying the same principles, it was not possible to fully capture the pulmonary PK of the acidic drug IND. The model predictions suggested a much faster increase in pulmonary concentrations than observed in vivo, resulting in overestimation of tissue concentrations over the first two hours. Figure 3 shows the observed and predicted concentration–time profiles for all four drugs.

### 3.3. Comparison with Total Lung Concentrations

**Figure 4:**
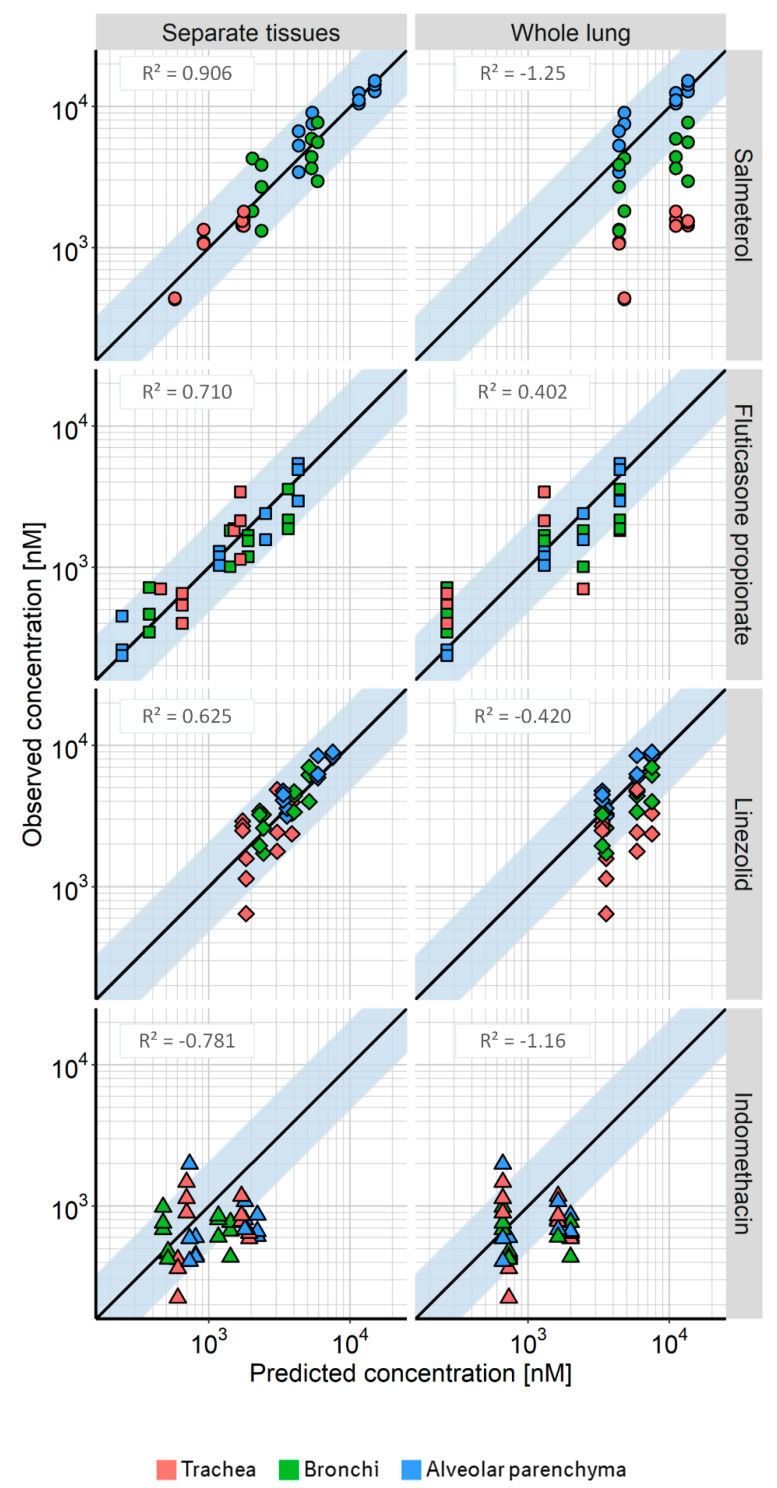
Goodness-of-fit plots of observed vs. predicted concentrations in the trachea (red), bronchi (green), and alveolar parenchyma (blue). Observed localized pulmonary concentrations plotted against (left) the predictions of concentrations in the separate pulmonary tissues; and (right) against predictions of total lung concentrations. Circles: salmeterol, squares: fluticasone propionate, diamonds: linezolid, triangles: indomethacin. The shaded area shows the two-fold error range.

The K_p_ values estimated based on total lung concentrations were similar to those found for the alveolar parenchyma (37.7 ± 2.7 for SAL, 6.21 ± 0.727 for FP, 0.781 ± 0.035 for LIN, and 0.347 ± 0.086 for IND), which was also the case for the blood flow estimate (11.6 ± 1.39 L/h/kg). The “whole lung” model was therefore able to describe concentrations in the alveolar region quite well. However, the concentration–time profiles in bronchi and trachea were not captured adequately (Figure 4; Supplementary Material 1, Figure S9).

Figure 4 shows the goodness-of-fit plots for both modelling of separate lung tissues and modelling of total lung concentrations compared to the observed concentrations in all three tissues. The model with separate compartments for trachea, bronchi, and alveolar parenchyma better described the data for all drugs, as shown by a better correlation (overall R^2^ of 0.879 for combined data of separate tissues across all drugs vs. 0.267 for combined data of whole lung predictions), as well as more predictions falling within the two-fold error range. The greatest improvement in terms of correlation was achieved for SAL, resulting in a R^2^ of 0.906 for the prediction of separate tissues, compared with an R^2^ of −1.25 for the prediction of total lung concentrations. The “whole lung” model tended to overpredict the concentrations in trachea and bronchi, which was especially evident for SAL and LIN.

### 3.4. Pulmonary Absorption Half-Lives

The pulmonary absorption half-lives (Table 2) were fastest for the alveolar parenchyma, followed by bronchi and trachea. Absorption half-lives calculated for the whole lung were similar to those in the alveolar region. The allometrically scaled values for humans were approximately four times larger than the absorption half-lives calculated for rats.

While these trends held true for all drugs, the absolute half-lives differed substantially between drugs. Both LIN and IND showed half-lives in the range of seconds, indicating rapid redistribution from the lung. In contrast, half-lives of FP and SAL ranged from minutes to hours, even in the rat, translating up to approximately five hours for SAL in the human trachea. Out of all drugs, FP showed the highest difference in half-life estimates between the different pulmonary tissues.

### 3.5. Time-Dependency of Tissue-to-Plasma Ratios

**Figure 5:**
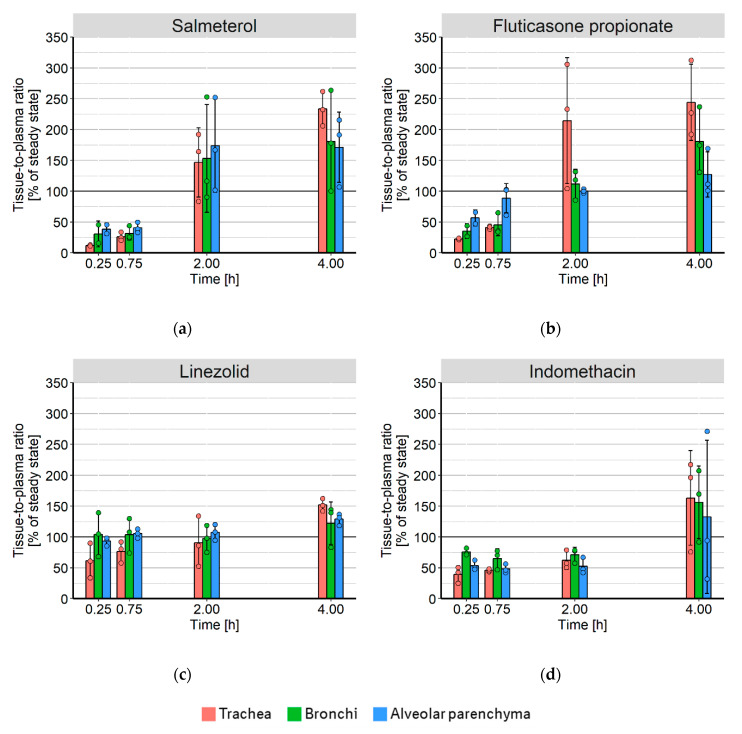
Observed tissue-to-plasma concentration ratios in the trachea (red), bronchi (green), and alveolar parenchyma (blue) as a percentage of steady state (the model estimate of K_p_) for (**a**) salmeterol, (**b**) fluticasone propionate, (**c**) linezolid, and (**d**) indomethacin. The bars represent the mean value including the standard deviation, the filled circles represent the individual data points.

The estimates for K_p_ for all drugs were further compared to the observed tissue-to-plasma ratios that could be extracted from the in vivo concentration measurements. Figure 5 shows the observed ratios for all drugs at the times of tissue sampling as a percentage of the tissue-to-plasma ratio at steady state (the model estimate for K_p_).

The equilibrium for LIN was achieved much faster than for SAL or FP, with only a slight delay shown in the trachea. The tissue-to-plasma ratios for LIN in the alveolar parenchyma and the bronchi were approximately stable after 15 min. The tissue-to-plasma ratios of SAL and FP were, however, not constant over time. Concentrations in the trachea did not reach steady state within the four-hour experiment, as the ratio between trachea and plasma concentrations was still rising between two and four hours. Tissue-to-plasma ratios of the alveolar parenchyma were stable after two hours but were higher than the expected ratio at steady state for SAL.

### 3.6. Effect of Distributional Delay on Plasma EC_50,Free_ Estimates of SAL

**Figure 6:**
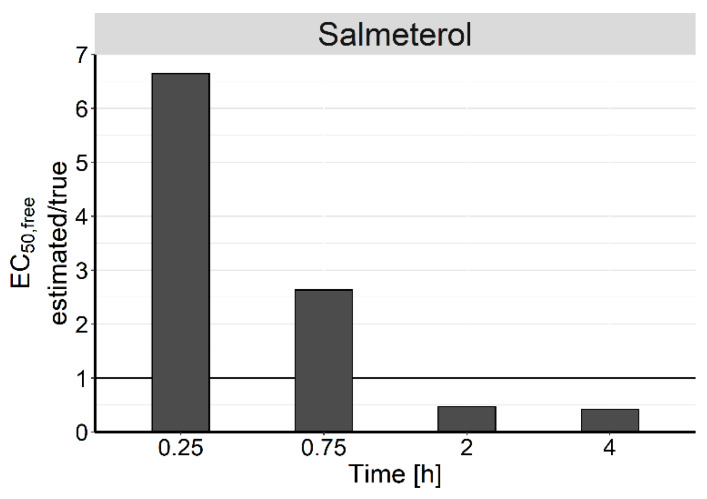
Plasma EC_50,free_ estimates of salmeterol normalized to the true EC_50_ value, determined at four different time points. Unbound bronchial tissue concentrations were assumed to be directly correlated to the effect.

The extended PK/PD model for SAL was used to simulate unbound plasma and bronchial concentrations and the predicted effect for a range of doses. The simulated effect and unbound plasma concentrations at 0.25 h, 0.75 h, 2 h, and 4 h were used for the determination of PD parameter estimates. Each investigated time point provided different estimates for the plasma EC_50,free_. When compared to the true value (0.0271 nM) the EC_50_ estimates determined at 0.25 h or 0.75 h overestimated the true EC_50,free_ by 6.62- and 2.62-fold, respectively (Figure 6). However, PD experiments at 2 h or 4 h resulted in an underestimation (0.467- and 0.418-fold) of the EC_50,free_, resulting in an approximately 16-fold divergence of estimates within the investigated timeframe. The ratio between the estimates and the true EC_50,free_ directly corresponded to the ratio between unbound concentrations in plasma and bronchi.

## 4. Discussion

The present study provides for the first time a systematic quantitative investigation of tissue retention in different pulmonary tissues for a set of four structurally diverse drugs. The retention for the trachea, bronchi, and the alveolar parenchyma of two neutral and one basic drugs was extensively investigated in vivo and was adequately described by a semi-mechanistic PK model. This model described the distribution into lung tissues considering physiological as well as drug driven differences, especially the fraction unbound in plasma, tissue-specific blood flows and K_p_ values. This investigation also highlights potential pitfalls derived from distributional delays to target tissues, when PK or PD predictions are based on single time point observations.

The available plasma PK data allowed the estimation of systemic PK parameters for FP, LIN, and IND. The plasma PK of SAL, FP, and LIN were best described by two-compartment models, which is in accordance with the literature [9,21,22]. While LIN PK in rats was previously only described by non-compartmental analysis [23], compartmental analyses of human PK typically do employ two compartments [24,25,26], which supports the use of a two-compartment PK model. IND concentrations in plasma were best captured by a one-compartment model, which is expected for acidic drugs with high plasma protein binding [27].

Preliminary PK studies for other drugs indicated that tissue sampling at three time points (after 1 h, 2 h, and 3 h) would not be sufficient to adequately quantify tracheal blood flow. Therefore, a stochastic simulation-estimation analysis was performed to select an adequate number and timing of tissue samples (Supplementary Material 1, Section 2). Based on these results, the studies to evaluate regional pulmonary disposition included four optimized time points for tissue sampling: Two of them were scheduled within the first hour (during the infusion) to capture the initial delay in tissue concentrations before the distribution equilibrium between plasma and the respective lung tissue is reached. As the blood flow is assumed the limiting parameter for this delay, these measurements were judged the most informative for the estimation of tissue blood flow. The last tissue samples were taken 2 and 4 h after start of the infusion. This 4-h time point was the latest possible time point due to the experimental setup, which was limited by the maximal tolerated time of anesthesia. The reason for the 4-h time point was to capture the tissue-to-plasma ratio at, or at least nearing distribution equilibrium to support the estimation of K_p_.

The here developed study design allowed an adequate estimation of the tissue-specific parameters (CV for most parameters <20%). Exceptions were the K_p_ values of IND and the bronchial blood flow, which were estimated with only slightly higher imprecision (see Table 1). In the case of IND, the suggested model was not able to accurately capture the pulmonary disposition (compare Figure 3d). This indicates that acidic drugs may provide different pulmonary PK characteristics compared to neutral and basic drugs and that the strictly perfusion-limited approach used in this investigation may not be appropriate for this drug type. However, it has to be noted that this has so far only been shown for one acidic drug. Further studies are necessary to confirm or refute similar behavior for different acids. As IND showed comparable permeability to the other tested drugs, permeability-limited kinetics were not deemed a reasonable explanation, and since binding to plasma proteins was accounted for, this was also deemed improbable as a cause. IND is known for being a substrate of active transport processes [28], so this might be a possible cause of the altered concentration–time profiles in pulmonary tissues.

The results of the in vivo PK studies revealed a time-dependency of tissue-to-plasma ratios. The extent of this varied for each drug-tissue combination. The semi-mechanistic PK model was able to describe this behavior based on the drug- and tissue-specific parameters. This was in accordance with the results of previous investigations [7] of regional localization of drugs in the lung, which also showed tissue-specific differences in tissue abundance over time. However, as Hamm et al. only qualitatively evaluated the relative abundance in bronchiolar and peripheral lung tissue at two time points (2 min and 30 min), a quantitative assessment of pulmonary distribution mechanisms was not possible based on their data. In this work, four time points and combination of the tissue distribution data of four drugs with varying physicochemical properties were necessary to achieve adequate estimates for both the pulmonary blood flows and tissue affinity.

The K_p_ values estimated in this study were vastly different between all four drugs. Out of all four drugs, SAL showed the highest tissue affinity (i.e., the highest estimated K_p_ values). While SAL is also quite lipophilic (logP of 2.5), this is likely due to the basic interactions with acidic phospholipids in the tissue (basic pKa of 9.8). This mechanism has been postulated before to be the major driver of tissue affinity for moderate-to-strong bases (basic pKa > 7) [18,29]. Furthermore, lysosomal trapping is known to play a role in the pulmonary tissue affinity of SAL [30,31], which is also attributable to SAL being a lipophilic base. The other drug showing moderate affinity to pulmonary tissues was FP, a neutral and highly lipophilic drug. For this type of drug, affinity is thought to be mainly determined by hydrophobic interactions with neutral lipids [32]. LIN, the other predominantly neutral drug (basic pKa of 1.8 [33,34]), did not show any increased affinity to pulmonary tissues in comparison to plasma and the K_p_ estimates were in a similar range than those reported by Slatter et al. [23]. Since LIN is less lipophilic than FP, and therefore does not have pronounced interactions with lipid structures in the tissue, this was not unexpected. IND showed the lowest affinity to pulmonary tissues, with tissue concentrations being more than two-fold lower than those in plasma. This may in part be due to its high affinity to plasma proteins, keeping the equilibrium on the plasma side. In addition, even though IND is highly lipophilic (logP of 4.08 [35]), the negative charge may prevent its partitioning into lipid membranes.

In addition to the variation in tissue affinity between the drugs, there were also differences in localized distribution. While FP and IND showed no discernable differences in affinity to the three lung tissues, both SAL and LIN showed higher affinity towards the alveolar parenchyma compared to the conducting airways. This behavior of SAL and FP is in line with the results of Hamm et al. [7]. Since FP and IND are both highly lipophilic and do not show differences in binding across different pulmonary tissues, the regional differences in tissue for both SAL and LIN are likely not caused by the hydrophobic tissue interactions. Instead, as the most pronounced difference was found for SAL, it is likely that the electrostatic interactions with acidic phospholipids or lysosomal trapping are important contributors to the observed difference in tissue exposure.

As a consequence of the observed regional differences in tissue affinity and blood flow, the semi-mechanistic PK model that included the separate tissue compartments described the local pulmonary concentrations of these drugs better than the model based on total lung concentrations (see Figure 4). The K_p_ estimates provided by the latter were close to those obtained for the alveolar parenchyma, which is not surprising, as this tissue is assumed to constitute about 80% of the total lung. Additionally, the total lung K_p_ estimates were in line with those found in the literature. The total lung K_p_ estimated for SAL (37.7 ± 2.7), FP (6.21 ± 0.73), LIN (0.781 ± 0.035), and IND (0.347 ± 0.086) were close to the values predicted by the Rodgers and Rowland method (32.5, 9.05, 0.706, and 0.228, respectively). The Rodgers and Rowland method is used to predict K_p_ values based on physicochemical drug properties (pKa, logP, blood-plasma ratio, and plasma protein binding), as well as physiological tissue composition [18,32]. In comparison with K_p_ values determined by the lung slice method [36], SAL showed slightly lower, (lung slice K_p_ between 46.5 and 64.8 [30,36]), and FP slightly higher affinity in our experiments (lung slice K_p_ 3.41 [36]). The overall good agreement indicates that analyses based on total lung concentrations and using K_p_ prediction methods like the Rodgers and Rowland method may be sufficient for predicting alveolar drug concentration–time profiles. However, if the drug’s target is located in the conducting airways, total lung concentrations may not be a meaningful surrogate, especially not for basic drugs.

Differences in pulmonary blood flow also lead to variations in concentrations between the different lung tissues. While the alveolar parenchyma is supplied by the pulmonary circulation (i.e., the total cardiac output), both the bronchi and the trachea are supplied by the systemic circulation. The estimate for alveolar blood flow in rats (10.6 L/h/kg) was lower than most values for cardiac output found in the literature (mean value: 15.1 L/h/kg), but was still within the reported range (10.3–20 L/h/kg [37,38,39,40,41]). The combined blood flows of bronchi and trachea amounted to 0.831 L/h/kg, which is higher compared to literature values for tracheobronchial blood flow (2.1% of cardiac output [12], 0.216–0.420 L/h/kg). However, the estimate for tracheal blood flow (0.054 L/h/kg) was more than two-fold lower than the weight-proportional part of the combined blood flow of 0.116 L/h/kg. To our knowledge, this is the first time that tracheal blood flow in rats has been estimated separately from the bronchial blood flow. Boger et al. [42] implemented generation-specific blood flow in their physiologically-based PK model as a function of airway diameter, based on an equation evaluated with dog data [43]. This relationship has not yet been validated for rats. However, when this equation is applied to the cumulative blood flow of bronchi and trachea found in this investigation, the resulting blood flow for the trachea would amount to 0.066 L/h/kg, which is in agreement with the actual estimate of 0.054 L/h/kg.

The alveolar parenchyma, as the best perfused tissue in the whole body [12], would typically not be expected to show a time delay compared to plasma. However, SAL and FP both showed delayed disposition in the alveolar region. By incorporating plasma protein binding (PPB) into the model this can be adequately described. We assume that the high PPB (>98%) is causative for the observed delayed disposition: Only a small portion of the drug in plasma is actually free to permeate, effectively slowing the partitioning into the lung tissues. The different combinations of tissue affinity, blood flow, and plasma protein binding lead to partitioning rates that are both tissue- and drug-specific. In general, partitioning is slow with high tissue affinity and increases with higher blood flow and fraction unbound. This held true for the trachea, which showed the slowest partitioning rates out of all three tissues.

The fact that the partitioning rate is also drug-specific makes it difficult to estimate the local tissue exposure of drugs based on plasma PK alone. Yet, since studying tissue distribution in humans is rarely possible, plasma PK is often used as a surrogate to infer on the concentration–effect relationship [4]. Our results showed that this approach might lead to very different estimates of EC_50,free_ depending on the time point of the PD experiment. Due to the dependency of partitioning rates on drug properties, the time-dependent variation in PD parameter estimates will differ for each drug. A comparison of drug potency based on plasma concentrations at a single time point would therefore not be advisable if no information on tissue distribution is available. To overcome some of the weaknesses, a thorough preclinical investigation of the target tissue distribution of new drugs seems meaningful. This definitely helps understanding the PK/PD relationships in animal models. It will also inform translation to humans as unspecific tissue binding seems to be essentially similar over a wide range of species, as shown for brain binding [44].

If tissue distribution data is available from pre-clinical experiments, attempts can be made to extrapolate the relationship to the human situation based on human physiology or allometric principles. In this investigation, pulmonary absorption half-lives were calculated from the model parameter estimates and subsequently extrapolated to human. Even if they cannot be viewed as full pulmonary half-lives, since they do not take the redistribution from plasma to the lung tissues into account, these absorption half-lives should be qualitatively comparable with absorption rate constants used for empirical models of oral inhalation without redistribution to the lung [45,46,47]. LIN and IND, both drugs that were not optimized for oral inhalation, showed very short absorption half-lives in the range of seconds, indicating a fast equilibrium between pulmonary tissues and plasma. This seems to be in agreement with the assumption that at least part of an orally inhaled drug shows “i.v. bolus-like” absorption after oral inhalation [45,48]. This could be true for the part of drug that is not retained in the lung or limited by slow dissolution. In contrast, SAL and FP show prolonged absorption half-lives in all investigated lung tissues. For SAL, the prolonged duration of effect achieved after oral inhalation has been associated with the pulmonary retention caused by high tissue affinity [2,8]. The pulmonary absorption half-lives calculated for FP ranged from 32 min in the alveolar region to 3.5 h in the trachea. However, most empirical PK models describing plasma PK of FP only identify a single absorption constant. This is in line with the common assumption that FP absorption from the lung is limited by the slow dissolution rather than the absorption itself. Reported absorption rate constants for FP were in the range of dissolution half-lives (3.85 h and 3.47 h, respectively [49,50]. Nevertheless, this investigation showed that even though the limiting factor may be the dissolution, FP still shows moderate retention in the conducting airways due to tissue affinity. There are also empirical models for other drugs that identified several parallel absorption processes [45,46,47]. For some of the drugs with rather high tissue affinity [46,47], this might be explained by different absorption rate constants depending on the lung region.

This study presents a method towards understanding localized pulmonary retention based on tissue affinity and pulmonary blood flow. However, it has to be noted that this study also showed that for drugs that are subject to permeability-limited kinetics or active transport processes, additional investigations beyond the here applied methods would be required. While permeability-limited kinetics may be implemented using in vitro permeability data, there is little quantitative information on expression and especially localization of transport proteins in the lung [51,52]. As most investigated drugs are lipophilic and/or well permeable, the influence of active drug transport on tissue partitioning at steady state was judged low [53]. Even though active transport processes were demonstrated in epithelial cells (i.e., between the tissue and the lining fluids) [51], the influence of the small volume of the epithelial lining fluid would be negligible on the measured pulmonary drug concentrations. Moreover, the here described approach may not be suitable for drugs that show non-linear tissue binding. For example, the sequestration into lysosomes was shown to be saturable at high unbound concentrations (>100 nM) [36]. As the highest unbound concentrations of SAL in this study were below 15 nM, tissue binding should still be within the linear range. However, this linearity might not hold true at very high inhaled doses, in which case the here described parameters could vary for different exposure levels in the lung. In contrast, other binding mechanisms relevant for the model drugs (partitioning into membranes, interactions with acidic phospholipids) are generally not saturable by commonly achieved concentrations [54]. The PK model in this study did not account for the residual blood content of the tissue samples. Even though the samples were obtained after exsanguination and rinsed with saline, especially the peripheral lung sample may still contain relevant amounts of residual blood (up to 28% [55]). The presence of residual blood in the alveolar parenchyma may be a reason for the comparably low estimate of alveolar blood flow. Furthermore, since lung tissue affinity is also drug-specific, the model cannot be directly adapted to drugs other than those used for the investigation. To achieve this, more work would have to be done to investigate which drug properties are relevant for differences in affinity between trachea, bronchi, and alveolar parenchyma, such as lipophilicity, charge, and affinity to plasma proteins. For a systematic analysis, more data for drugs with different physico-chemical characteristics would be needed. Moreover, an examination of the regional tissue composition with regard to lipid types and lysosome content [56,57,58,59] would be very helpful. This data could be used to evaluate if the K_p_ prediction models such as Rodgers and Rowland can be used to extrapolate the model to different drugs. Additionally, the here presented model could be further extended to oral inhalation. By developing the model on drug PK after intravenous administration, the distribution process between plasma and lung tissue could be investigated separately from other relevant processes. However, to make the model applicable to oral inhalation, the PK processes specific to oral inhalation, like deposition patterns, mucociliary clearance, pulmonary dissolution, and absorption from the epithelial lining fluid [4] would have to be implemented. With these processes included, this type of model could be used to assess advantages and disadvantages of pulmonary drug delivery depending on physico-chemical drug characteristics and the target location within the lung.

## 5. Conclusions

In summary, this manuscript introduces a semi-mechanistic model to describe regional pulmonary tissue retention based on physiological and drug-specific parameters. The model successfully captured the pulmonary disposition of the investigated neutral and basic drugs. Additional investigations are required; especially regarding acidic drugs since further PK processes in the lung seem to be relevant. The in vivo studies showed that structural differences between the conducting airways and the alveolar parenchyma resulted in different tissue affinity and retention times for basic drugs. Considering whole lung concentrations was in most cases not representative of the conducting airways, representing the target site for many locally-acting orally inhaled drugs. The estimated pulmonary blood flows for alveolar parenchyma and cumulative blood flow for both trachea and bronchi were in accordance with literature values. This supports the separate tissue retention estimates for trachea and bronchi, which were, to the knowledge of the authors, reported for the first time in this study.

The high tissue affinity and extensive protein binding of SAL, in combination with low blood flow resulted in marked distributional delay in the conducting airways. Further investigations on the estimation of PD parameters from a single time point revealed that, under these circumstances, plasma concentrations are no valid surrogate for pulmonary target-site concentrations. This work highlights the importance of being aware of the physiologic differences between lung tissues and their impact on local PK, as well as the use of time-resolved PK data combined with model-based approaches to gain a better understanding of local lung retention and local efficacy to guide identification of drugs for lung diseases.

## Figures and Tables

**Table 1:** Model parameters (% CV).

Parameter	Unit	Salmeterol	Fluticasone Propionate	Linezolid	Indomethacin
CL	L·h^−1^·kg^−1^	3.86 (6.07)	3.37 (3.42)	0.279 (2.22)	0.0691 (11.0)
V_C_	L·kg^−1^	0.123 (16.2)	0.223 (56.0)	0.320 (19.7)	0.154 (5.27)
Q	L·h^−1^·kg^−1^	3.24 (21.1)	4.72 (12.8)	2.79 (34.9)	-
V_P_	L·kg^−1^	3.77 (14.6)	2.41 (8.18)	0.628 (9.96)	-
K_p,T_	-	6.52 (7.04)	5.21 (16.9)	0.404 (11.2)	0.356 (20.2)
K_p,B_	-	18.6 (12.2)	6.64 (13.2)	0.534 (6.52)	0.249 (16.0)
K_p,A_	-	39.3 (8.10)	5.84 (10.6)	0.785 (5.11)	0.384 (35.5)
Q_T_^1^	L·h^−1^·kg^−1^	0.054 (14.3)			
Q_B_^1^	L·h^−1^·kg^−1^	0.777 (21.5)			
Q_A_^1^	L·h^−1^·kg^−1^	10.6 (10.7)			

Abbreviations are provided in Figure 2. ^1^ Blood flows were estimated simultaneously for all drugs.

**Table 2:** Pulmonary absorption half-lives (t_½_) ^1^ derived from model parameters.

Drug	Tissue	t_½_ (Rat)	t_½_ (Human) ^2^
Salmeterol	Trachea	1.2 h	4.75 h
	Bronchi	57 min	3.77 h
	Alveolar	45 min	2.91 h
	Full lung	48.3 min	3.20 h
Fluticasone propionate	Trachea	52.4 min	3.48 h
	Bronchi	18.6 min	1.23 h
	Alveolar	5.99 min	23.8 min
	Full lung	7.28 min	28.9 min
Linezolid	Trachea	0.5 s	2.0 s
	Bronchi	0.2 s	0.8 s
	Alveolar	0.1 s	0.4 s
	Full lung	0.1 s	0.5 s
Indomethacin	Trachea	14 s	55 s
	Bronchi	2.7 s	11 s
	Alveolar	1.5 s	6.0 s
	Full lung	1.6 s	6.2 s

^1^ Unidirectional flow from the lung compartments to the central compartment. ^2^ Human half-lives were allometrically scaled from rat values.

## Supplementary Materials

The following are available online at https://www.mdpi.com/1999-4923/12/5/408/s1, Supplementary material 1: Stochastic simulation-estimation (SSE) study, Bioanalysis, Figure S1: Chemical structures of the four model drugs, Table S1: Physicochemical characteristics of the model drugs, Figure S2: Structure of the model used in the SSE study, Table S2: Parameters used for the simulation of concentration–time profiles for the SSE study, Table S3: Bias (mean) and coefficient of variation (CV) of the parameter estimates from the SSE study, Figure S3: Parameter estimate distribution for the initial and optimized study designs, Table S4: Study design and sample weights, Figure S4–S8: Calibration curves and exemplary LC-MS chromatograms, Figure S9: Concentration–time profiles predicted for plasma (black) and total lung in comparison to the observed concentrations in the separate lung tissues, Supplementary Material 2: Phoenix model code.

## References

[1] Kelly H.W. (1998). Establishing a therapeutic index for the inhaled corticosteroids: Part i: Pharmacokinetic/pharmacodynamic comparison of the inhaled corticosteroids. J. Allergy Clin. Immunol..

[2] Lombardi D., Cuenoud B., Krämer S.D. (2009). Lipid membrane interactions of indacaterol and salmeterol: Do they influence their pharmacological properties?. Eur. J. Pharm. Sci..

[3] Begg M., Edwards C.D., Hamblin J.N., Pefani E., Wilson R., Gilbert J., Vitulli G., Mallett D., Morrell J., Hingle M.I. (2019). Translation of inhaled drug optimization strategies into clinical pharmacokinetics and pharmacodynamics using gsk2292767a, a novel inhaled phosphoinositide 3-kinase δ inhibitor. J. Pharmacol. Exp. Ther..

[4] Borghardt J.M., Kloft C., Sharma A. (2018). Inhaled therapy in respiratory disease: The complex interplay of pulmonary kinetic processes. Can. Respir. J..

[5] Mutlu G.M., Factor P. (2008). Alveolar epithelial β2-adrenergic receptors. Am. J. Respir. Cell Mol. Biol..

[6] Backstrom E., Hamm G., Nilsson A., Fihn B.M., Strittmatter N., Andren P., Goodwin R.J.A., Friden M. (2018). Uncovering the regional localization of inhaled salmeterol retention in the lung. Drug Deliv..

[7] Hamm G.R., Bäckström E., Brülls M., Nilsson A., Strittmatter N., Andrén P.E., Grime K., Fridén M., Goodwin R.J. (2020). Revealing the regional localization and differential lung retention of inhaled compounds by mass spectrometry imaging. J. Aerosol Med. Pulm. Drug Deliv..

[8] Hendrickx R., Bergström E.L., Janzén D.L.I., Fridén M., Eriksson U., Grime K., Ferguson D. (2018). Translational model to predict pulmonary pharmacokinetics and efficacy in man for inhaled bronchodilators. CPT Pharmacomet. Syst. Pharmacol..

[9] Weber B., Hochhaus G. (2013). A pharmacokinetic simulation tool for inhaled corticosteroids. AAPS J..

[10] Iqbal K., Broeker A., Nowak H., Rahmel T., Nussbaumer-Pröll A., Österreicher Z., Zeitlinger M., Wicha S. (2020). A pharmacometric approach to define target site-specific breakpoints for bacterial killing and resistance suppression integrating microdialysis, time-kill curves and heteroresistance data: A case study with moxifloxacin. Clin. Microbiol. Infect..

[11] Boger E., Evans N., Chappell M., Lundqvist A., Ewing P., Wigenborg A., Fridén M. (2016). Systems pharmacology approach for prediction of pulmonary and systemic pharmacokinetics and receptor occupancy of inhaled drugs. CPT Pharmacomet. Syst. Pharmacol..

[12] Brown R.P., Delp M.D., Lindstedt S.L., Rhomberg L.R., Beliles R.P. (1997). Physiological parameter values for physiologically based pharmacokinetic models. Toxicol. Ind. Health.

[13] Anderson P.J., Zhou X., Breen P., Gann L., Logsdon T.W., Compadre C.M., Hiller F.C. (1998). Pharmacokinetics of (r, s)-albuterol after aerosol inhalation in healthy adult volunteers. J. Pharm. Sci..

[14] Minto C., Li B., Tattam B., Brown K., Seale J.P., Donnelly R. (2000). Pharmacokinetics of epimeric budesonide and fluticasone propionate after repeat dose inhalation–intersubject variability in systemic absorption from the lung. Br. J. Clin. Pharmacol..

[15] Rohatagi S., Arya V., Zech K., Nave R., Hochhaus G., Jensen B., Barrett J. (2003). Population pharmacokinetics and pharmacodynamics of ciclesonide. J. Clin. Pharmacol..

[16] Ting L., Aksenov S., Bhansali S., Ramakrishna R., Tang P., Geller D. (2014). Population pharmacokinetics of inhaled tobramycin powder in cystic fibrosis patients. CPT Pharmacomet. Syst. Pharmacol..

[17] Wu K., Goyal N., Stark J.G., Hochhaus G. (2008). Evaluation of the administration time effect on the cumulative cortisol suppression and cumulative lymphocytes suppression for once-daily inhaled corticosteroids: A population modeling/simulation approach. J. Clin. Pharmacol..

[18] Rodgers T., Leahy D., Rowland M. (2005). Physiologically based pharmacokinetic modeling 1: Predicting the tissue distribution of moderate-to-strong bases. J. Pharm. Sci..

[19] Johnson M., Butchers P., Coleman R., Nials A., Strong P., Summer M., Vardey C., Whelan C. (1993). The pharmacology of salmeterol. Life Sci..

[20] R Core Team (2016). A Language and Environment for Statistical Computing, 3.3.2.

[21] Bhagwat S., Schilling U., Chen M.J., Wei X., Delvadia R., Absar M., Saluja B., Hochhaus G. (2017). Predicting pulmonary pharmacokinetics from in vitro properties of dry powder inhalers. Pharm. Res..

[22] Soulele K., Macheras P., Karalis V. (2017). Pharmacokinetic analysis of inhaled salmeterol in asthma patients: Evidence from two dry powder inhalers. Biopharm. Drug Dispos..

[23] Slatter J.G., Adams L.A., Bush E.C., Chiba K., Daley-Yates P.T., Feenstra K.L., Koike S., Ozawa N., Peng G.W., Sams J.P. (2002). Pharmacokinetics, toxicokinetics, distribution, metabolism and excretion of linezolid in mouse, rat and dog. Xenobiotica.

[24] Beringer P., Nguyen M., Hoem N., Louie S., Gill M., Gurevitch M., Wong-Beringer A. (2005). Absolute bioavailability and pharmacokinetics of linezolid in hospitalized patients given enteral feedings. Antimicrob. Agents Chemother..

[25] Bhalodi A.A., Papasavas P.K., Tishler D.S., Nicolau D.P., Kuti J.L. (2013). Pharmacokinetics of intravenous linezolid in moderately to morbidly obese adults. Antimicrob. Agents Chemother..

[26] Plock N., Buerger C., Joukhadar C., Kljucar S., Kloft C. (2007). Does linezolid inhibit its own metabolism?—population pharmacokinetics as a tool to explain the observed nonlinearity in both healthy volunteers and septic patients. Drug Metab. Dispos..

[27] Pilari S., Huisinga W. (2010). Lumping of physiologically-based pharmacokinetic models and a mechanistic derivation of classical compartmental models. J. Pharmacokinet. Pharmacodyn..

[28] Kouzuki H., Suzuki H., Sugiyama Y. (2000). Pharmacokinetic study of the hepatobiliary transport of indomethacin. Pharm. Res..

[29] Rodgers T., Leahy D., Rowland M. (2005). Tissue distribution of basic drugs: Accounting for enantiomeric, compound and regional differences amongst β-blocking drugs in rat. J. Pharm. Sci..

[30] Bäckström E., Boger E., Lundqvist A., Hammarlund-Udenaes M., Fridén M. (2016). Lung retention by lysosomal trapping of inhaled drugs can be predicted in vitro with lung slices. J. Pharm. Sci..

[31] Kazmi F., Hensley T., Pope C., Funk R.S., Loewen G.J., Buckley D.B., Parkinson A. (2013). Lysosomal sequestration (trapping) of lipophilic amine (cationic amphiphilic) drugs in immortalized human hepatocytes (fa2n-4 cells). Drug Metab. Dispos..

[32] Rodgers T., Rowland M. (2006). Physiologically based pharmacokinetic modelling 2: Predicting the tissue distribution of acids, very weak bases, neutrals and zwitterions. J. Pharm. Sci..

[33] Chiang P.-C., Hu Y. (2009). Simultaneous determination of logd, logp, and pka of drugs by using a reverse phase hplc coupled with a 96-well plate auto injector. Comb. Chem. High Throughput Screen..

[34] Taylor R., Sunderland B., Luna G., Czarniak P. (2017). Evaluation of the stability of linezolid in aqueous solution and commonly used intravenous fluids. Drug Des. Dev. Ther..

[35] Inagi T., Muramatsu T., Nagai H., Terada H. (1981). Mechanism of indomethacin partition between n-octanol and water. Chem. Pharm. Bull..

[36] Bäckström E., Lundqvist A., Boger E., Svanberg P., Ewing P., Hammarlund-Udenaes M., Fridén M. (2016). Development of a novel lung slice methodology for profiling of inhaled compounds. J. Pharm. Sci..

[37] Coleman T.G. (1974). Cardiac output by dye dilution in the conscious rat. J. Appl. Physiol..

[38] Delp M., Manning R., Bruckner J., Armstrong R. (1991). Distribution of cardiac output during diurnal changes of activity in rats. Am. J. Physiol.-Heart Circ. Physiol..

[39] Hachamovitch R., Wicker P., Capasso J.M., Anversa P. (1989). Alterations of coronary blood flow and reserve with aging in fischer 344 rats. Am. J. Physiol.-Heart Circ. Physiol..

[40] Tsuchiya M., Ferrone R.A., Walsh G.M., Frohlich E.D. (1978). Regional blood flows measured in conscious rats by combined fick and microsphere methods. Am. J. Physiol.-Heart Circ. Physiol..

[41] Walsh G.M., Tsuchiya M., Frohlich E.D. (1976). Direct fick application for measurement of cardiac output in rat. J. Appl. Physiol..

[42] Boger E., Fridén M. (2019). Physiologically based pharmacokinetic/pharmacodynamic modeling accurately predicts the better bronchodilatory effect of inhaled versus oral salbutamol dosage forms. J. Aerosol. Med. Pulm. Drug Deliv..

[43] Bernard S.L., Glenny R.W., Polissar N.L., Luchtel D.L., Lakshminarayan S. (1996). Distribution of pulmonary and bronchial blood supply to airways measured by fluorescent microspheres. J. Appl. Physiol..

[44] Di L., Umland J.P., Chang G., Huang Y., Lin Z., Scott D.O., Troutman M.D., Liston T.E. (2011). Species independence in brain tissue binding using brain homogenates. Drug Metab. Dispos..

[45] Bartels C., Looby M., Sechaud R., Kaiser G. (2013). Determination of the pharmacokinetics of glycopyrronium in the lung using a population pharmacokinetic modelling approach. Br. J. Clin. Pharmacol..

[46] Borghardt J.M., Weber B., Staab A., Kunz C., Formella S., Kloft C. (2016). Investigating pulmonary and systemic pharmacokinetics of inhaled olodaterol in healthy volunteers using a population pharmacokinetic approach. Br. J. Clin. Pharmacol..

[47] Melin J., Prothon S., Kloft C., Cleton A., Amilon C., Jorup C., Bäckman P., Olsson B., Hamrén U.W. (2017). Pharmacokinetics of the inhaled selective glucocorticoid receptor modulator azd5423 following inhalation using different devices. AAPS J..

[48] Soulele K., Macheras P., Silvestro L., Savu S.R., Karalis V. (2015). Population pharmacokinetics of fluticasone propionate/salmeterol using two different dry powder inhalers. Eur. J. Pharm. Sci..

[49] Krishnaswami S., Hochhaus G., Möllmann H., Barth J., Derendorf H. (2005). Interpretation of absorption rate data for inhaled fluticasone propionate obtained in compartmental pharmacokinetic modeling. Int. J. Clin. Pharmacol. Ther..

[50] Rohrschneider M., Bhagwat S., Krampe R., Michler V., Breitkreutz J.r., Hochhaus G.n. (2015). Evaluation of the transwell system for characterization of dissolution behavior of inhalation drugs: Effects of membrane and surfactant. Mol. Pharm..

[51] Bosquillon C. (2010). Drug transporters in the lung--do they play a role in the biopharmaceutics of inhaled drugs?. J. Pharm. Sci..

[52] Sakamoto A., Matsumaru T., Yamamura N., Uchida Y., Tachikawa M., Ohtsuki S., Terasaki T. (2013). Quantitative expression of human drug transporter proteins in lung tissues: Analysis of regional, gender, and interindividual differences by liquid chromatography–tandem mass spectrometry. J. Pharm. Sci..

[53] Horvath G., Mendes E.S., Schmid N., Schmid A., Conner G.E., Salathe M., Wanner A. (2007). The effect of corticosteroids on the disposal of long-acting β2-agonists by airway smooth muscle cells. J. Allergy Clin. Immunol..

[54] Hallifax D., Houston J.B. (2007). Saturable uptake of lipophilic amine drugs into isolated hepatocytes: Mechanisms and consequences for quantitative clearance prediction. Drug Metab. Dispos..

[55] Oeff K., König A. (1955). Das blutvolumen einiger rattenorgane und ihre restblutmenge nach entbluten bzw. Durchspülung. Bestimmung mit p32-markierten erythrocyten. Naunyn-Schmiedebergs Archiv für Experimentelle Pathologie und Pharmakologie.

[56] Bligh E.G., Dyer W.J. (1959). A rapid method of total lipid extraction and purification. Can. J. Biochem. Physiol..

[57] Folch J., Lees M., Sloane Stanley G.H. (1957). A simple method for the isolation and purification of total lipides from animal tissues. J. Biol. Chem..

[58] Koivusalo M., Haimi P., Heikinheimo L., Kostiainen R., Somerharju P. (2001). Quantitative determination of phospholipid compositions by esi-ms: Effects of acyl chain length, unsaturation, and lipid concentration on instrument response. J. Lipid Res..

[59] Pulfer M., Murphy R.C. (2003). Electrospray mass spectrometry of phospholipids. Mass. Spectrom. Rev..

