# Supplementary material for: Towards a Quantitative Mechanistic Understanding of Localized Pulmonary Tissue Retention—A Combined In Vivo/In Silico Approach Based on Four Model Drugs
Source: Pharmaceutics. 2020 Apr 29;12(5):408. doi: 10.3390/pharmaceutics12050408 (PMC7284631; doi:10.3390/pharmaceutics12050408)
Supplement: Supplementary file 1 [file pharmaceutics-12-00408-s001.zip › Supplementary_material_1.pdf]

Supplementary material 1

1. Structures and key properties of model drugs

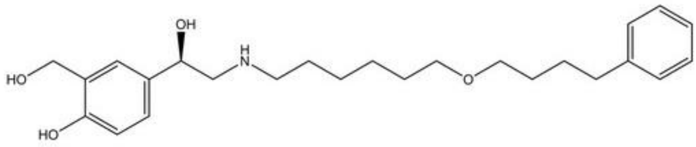

(**a**)

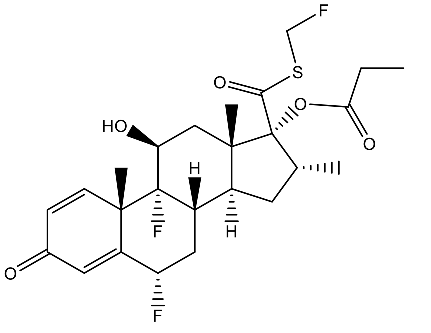

(**b**)

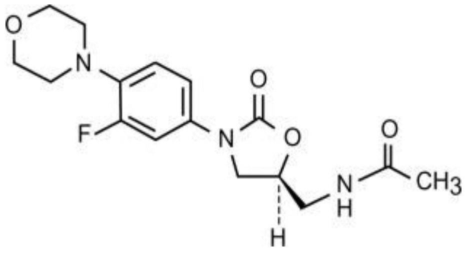

(**c**)

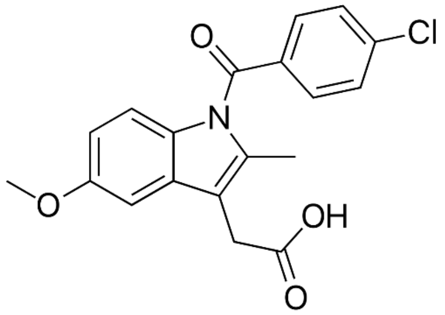

(**d**)

Figure S1. Chemical structures of the four model drugs. (a) Salmeterol; (b) fluticasone propionate; (c) linezolid; (d) indomethacin.

Table S1. Physicochemical characteristics of the model drugs.

Drug	Molecular weight [g/mol]	LogP	pKa	PPB rat	B:P	Permeability [10^{-6} cm/s]
**SAL**	604 (Base: 416)	2.5	9.8 (basic)	98.6%	1.70	14.0
**FP**	501	4.37	NA	98.5%	0.90	49.4
**LIN**	337	0.59	1.8 (basic)[b,c]	26.8%	0.93	20.4
**IND**	358	4.08[a]	4.5[d]	97.6%	NA	22.0

SAL: salmeterol, FP: fluticasone propionate, LIN: linezolid, IND: indomethacin, LogP: octanol/water partition coefficient, PPB: plasma protein binding, B:P: blood-to-plasma ratio. [a] Inagi et al. [1], [b] Taylor et al. [2], [c] Chiang et al. [3], [d] Budavari et al. [4]. All other values were measured in-house. Permeability was measured in a Caco-2 cell assay. The methods for the protein binding and permeability assays can be found elsewhere [5].

2. Study planning – Stochastic simulation-estimation (SSE) study

2.1. Methods

A stochastic simulation and estimation approach (SSE) was performed in R (Version 3.3.2) to optimize the design of the intravenous lung distribution studies for the simultaneous estimation of pulmonary blood flows and K_P values. The pharmacokinetic (PK) model used for this analysis consisted of two systemic compartments and one tissue compartment representing the trachea (Figure S2).

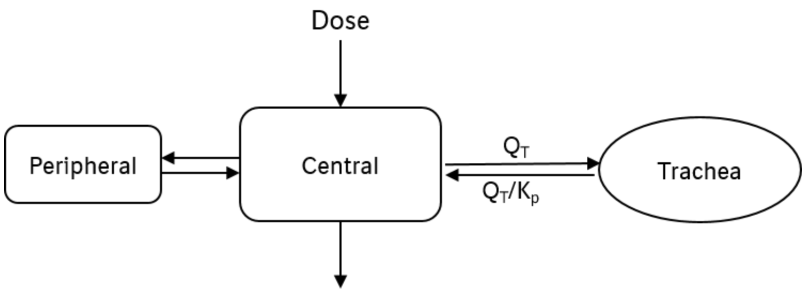

Figure S2. Structure of the model used in the SSE study. Q_T: tracheal blood flow; K_P: tissue-to-plasma partition coefficient.

PK parameters were derived from a preliminary cocktail PK study and can be found in Table S2. The weight of the trachea was based on measured weights from previous studies (0.0002 kg/kg bodyweight) the initial value for tracheal blood flow was scaled from literature data (bronchial circulation adjusted for trachea weight: 0.06 L/h/kg [ref Brown]). The K_P was set to 70, assuming that at least one drug with high tissue affinity would be included in the study, facilitating the estimation of blood flow.

Table S2. Parameters used for the simulation of concentration-time profiles.

Parameter	CL	V	Q	V2	Q_T	K_P	Prop. error
**Unit**	$\frac{L}{h \cdot kg}$	$\frac{L}{kg}$	$\frac{L}{h \cdot kg}$	$\frac{L}{kg}$	$\frac{L}{h \cdot kg}$	-	-
**Value**	8.69	4.95	3.68	9.49	0.06	70	0.112

CL: systemic clearance, V: central volume of distribution (Vd), Q: intercompartmental clearance, V2: peripheral Vd; Q_T: blood flow to the trachea; K_P: tissue-to-plasma partition coefficient, Prop. error: proportional residual variability.

One thousand sets of simulated plasma and tissue concentration-time profiles were generated for two different sampling schemes. The first sampling scheme matched the one employed in the first study, with tissue samples taken after 1, 2, and 3 hours after start of the one-hour infusion (Study design I, 3 samples per time point). The second design included four time points of tissue samples (0.25 h, 0.75 h, 2 h, and 4 h after start of the one-hour infusion; study design II, 4 samples per time point). Each of these simulated datasets was used again as the basis for the simultaneous estimation of systemic and tissue-specific parameters. The distribution of resulting parameter estimates was further evaluated to assess identifiability, bias and imprecision of the model parameters.
Relative bias of the mean and the coefficient of variation were calculated as follows:

$$\text{Bias} = \frac{\mu_{estimates} - true}{true} \cdot 100\%, \quad \text{(S1)}$$

with $\mu_{estimates}$ being the mean value of all 1000 estimates for a given parameter and true as the true value used for data simulation. The coefficient of variation (CV) was calculated by dividing the standard deviation of estimates ($\sigma_{estimates}$, n =1000)

$$\sigma_{estimates} = \sqrt{\frac{\sum(estimate - true)^2}{n-1}}, \quad \text{(S2)}$$

by the underlying true value and multiplying the result with 100:

$$\text{CV} = \frac{\sigma_{estimates}}{true} \cdot 100\%. \quad \text{(S3)}$$

2.2. Results

The SSE studies indicated an adequate estimation of Kp (24.5 %CV, bias 0.113%), whereas an accurate estimation of lung tissue perfusion was not possible (1117% CV, bias 369%) with study design I, see Supplementary table 3. With study design II, the accuracy and bias of the blood flow estimation was much better (37.0% CV, bias 6.33%) without losing any precision regarding the K_P.

Table S3. Bias (mean) and coefficient of variation (CV) of the parameter estimates from the SSE study.

Study design	Parameter	CL	V	Q	V2	Q_T	K_P
		$\frac{L}{h \cdot kg}$	$\frac{L}{kg}$	$\frac{L}{h \cdot kg}$	$\frac{L}{kg}$	$\frac{L}{h \cdot kg}$	-
I	**Bias, %**	-0.677	-0.470	-1.86	4.63	369	0.113
	**CV, %**	7.87	16.7	20.0	30.5	1117	24.5
II	**Bias, %**	-0.310	-0.264	0.696	4.08	6.33	0.133
	**CV, %**	6.18	12.7	14.4	30.7	37.0	9.64

CL: systemic clearance, V: central volume of distribution (Vd), Q: intercompartmental clearance, V2: peripheral Vd; Q_T: blood flow to the trachea; K_P: tissue-to-plasma partition coefficient.

A boxplot of the parameter estimate distribution around the true value can be found in Figure S3.

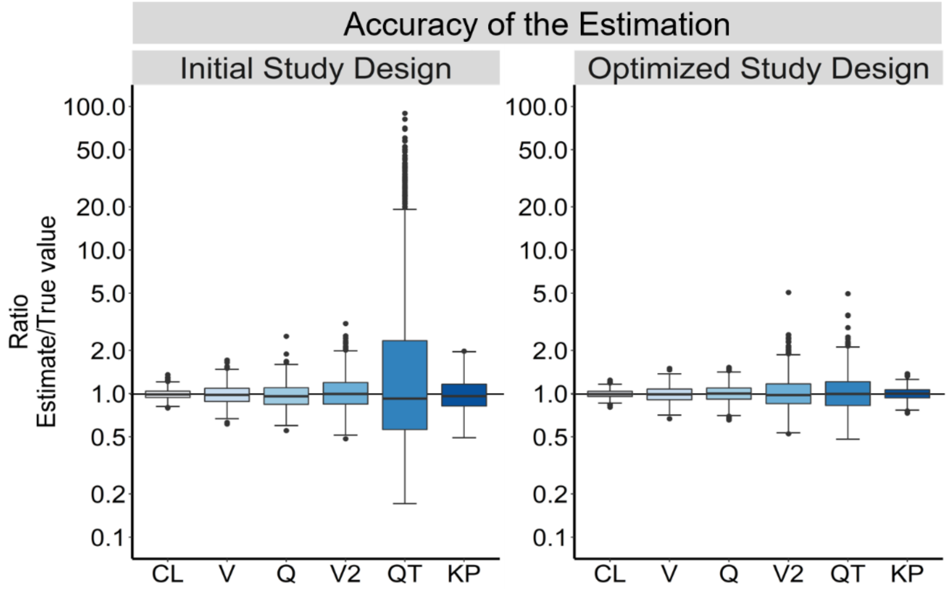

Figure S3. Parameter estimate distribution for the initial and optimized study designs (Study design I and II, respectively). The values are normalized to the true value used for the simulation of PK data. CL: systemic clearance, V: central volume of distribution (Vd), Q: intercompartmental clearance, V2: peripheral Vd; QT: blood flow to the trachea; KP: tissue-to-plasma partitioning coefficient.

Based on these results, estimation of localized pulmonary blood flow was deemed possible with the new study design II. However, the coefficient of variation of 37% was still quite high. This was addressed by the decision to estimate the blood flow simultaneously across all four model drugs to provide a richer dataset. Due to this, the number of tissue samples per time point could be reduced to three again.

3. Sampling scheme & Sample weights

See Table S4.

4. Additional PK study for salmeterol

The additional PK study used for the estimation of systemic PK of salmeterol was performed under the same overall conditions as the other *in vivo* studies. Four rats received salmeterol as part of a cocktail as an intravenous bolus dose of 4.81 µmol /kg. Blood samples (volume 100 µL) were collected at 0.083 h, 0.25 h, 0.5 h, 1.0 h, 2.0 h, 3.0 h, and 4.0 h.

5. Bioanalysis

5.1. Sample Preparation

Internal standards BI-1052 (internal source, 100 nM), indomethacin-d4 (Biomol GmbH, 500 nM), fluticasone propionate-d5 (Santa Cruz Biotechnology Inc, 100 nM), and linezolid-d3 (Biomol GmbH, 10 nM) were used for salmeterol (SAL), indomethacin (IND), fluticasone propionate (FP) and linezolid (LIN), respectively. After protein precipitation was performed by addition of acetonitrile, the samples were centrifuged at 50000 g for 10 minutes and the supernatant was injected into the LC system.

5.2. LC-MS Analysis

SAL was analysed using a Prodigy 5u ODS3 100A, 50 x 2 mm (Phenomenex, Torrance, CA, USA) at room temperature. Solvent A was consisting of 0.02% formic acid and solvent B of acetonitrile. The LC-gradient started at 5% solvent B and increased from 0.5 – 2.0 min to 90% at a flow rate of 500 µL/min. From 2.0 to 3.5 min it was maintained at 90%. The column was conditioned for 0.5 min at 5% solvent B. The injection volume was 5 µL. FP, LIN and IND were analyzed using a YMC Hydrosphere C18, 2.1 x 33 mm, 3 µm (YMC Co. Ltd., Kyoto, Japan) at room temperature. Solvent A was consisting of 0.1% formic acid and solvent B of acetonitrile and methanol (1:1) supplemented with 0.1% formic acid. The LC-gradient started at 5% solvent B and increased from 0.5 – 2.0 min to 50% at a flow rate of 500 µL/min. From 2.0 to 3.5 min it was maintained at 50%. The column was conditioned for 0.5 min at 5% solvent B. The injection volumes were 5 µL for FP and IND, and 2 µL for LIN.

A Thermo ScientificTM TSQ AltisTM triple quadrupole mass spectrometer was used for the analysis of SAL and IND, while a SCIEX QTRAP 6500 was used for FP and LIN. Both mass spectrometers were operated in positive mode.

Table S5. Mass spectrometry parameters.

Drug	Q1	Q3	CE
Salmeterol	416.3	91.1	55
BI-1052	453.1	275.1	27
Fluticasone propionate	501.2	313.2	19
Fluticasone propionate-d_5	506.2	313.2	19
Linezolid	338.1	296.2	25
Linezolid-d_3	341.1	297.2	25
Indomethacin	358.4	139.1	18
Indomethacin-d_4	362.4	143.1	20

Multiple quality controls were carried out to assess the method robustness. Separate calibrations were performed for the investigated drugs and their respective internal standards. The concentrations used for calibration ranged from 1 to 15000 nM for SAL (several calibration batches), and 10 to 20000 nM for FP, LIN and IND. All measured samples fell within the calibration range. Quality controls were performed at 20, 200, and 10000 nM for SAL, and at 20, 200, and 15000 nM for the other drugs.

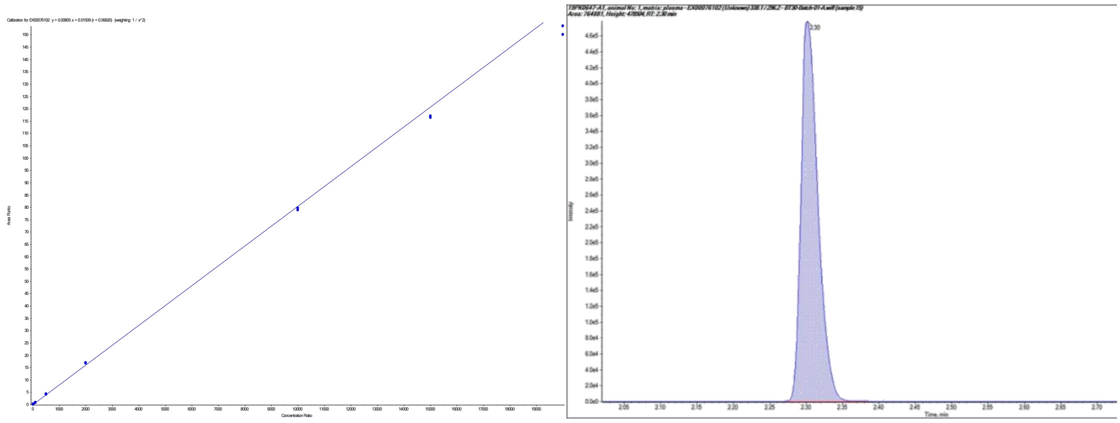

Figure S4. Calibration curve for LIN (left) and LC-MS chromatogram of an exemplary plasma sample (right).

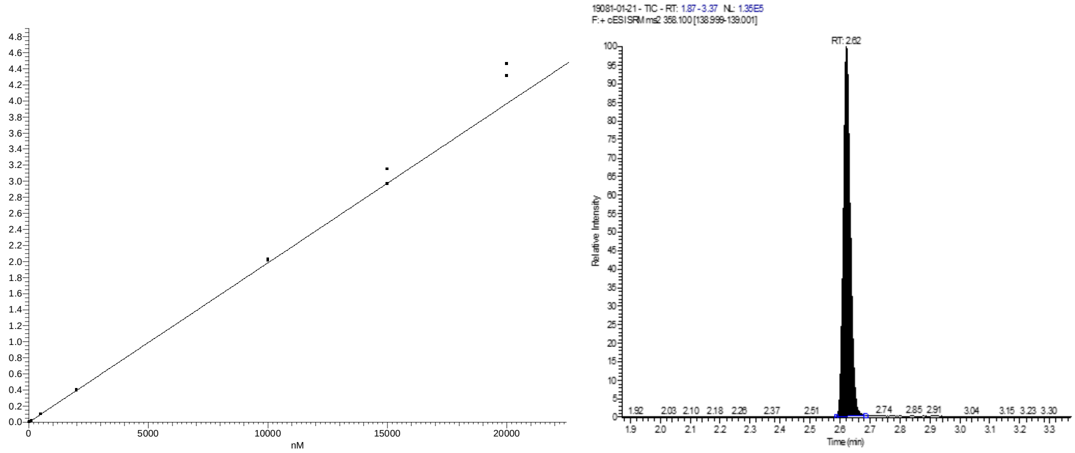

Figure S5. Calibration curve for IND (left) and LC-MS chromatogram of an exemplary plasma sample (right).

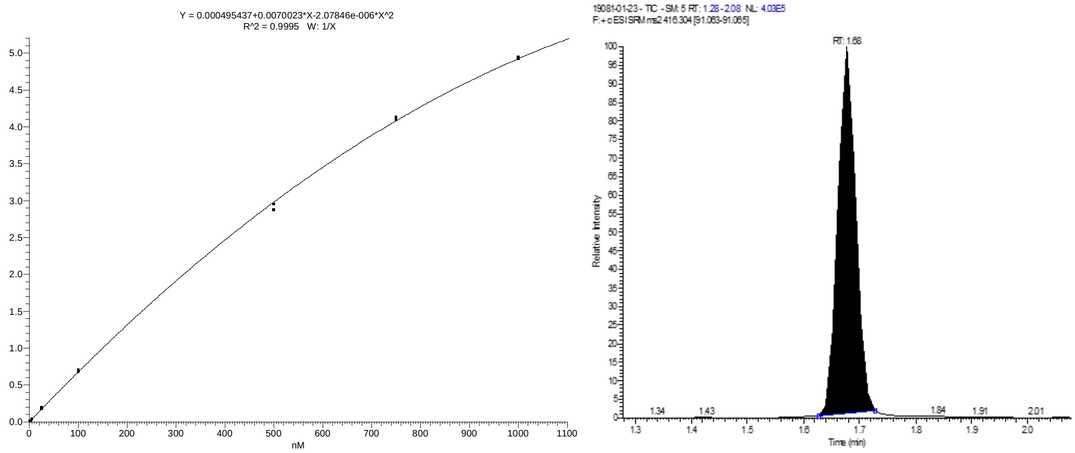

Figure S6. Calibration curve for SAL (left) and LC-MS chromatogram of an exemplary plasma sample (right).

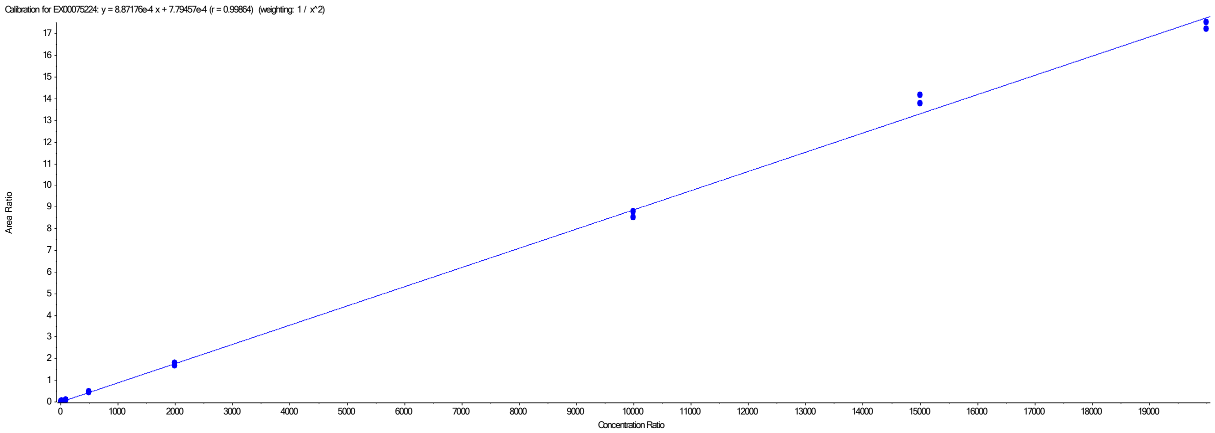

Figure S7. Calibration curve of FP.

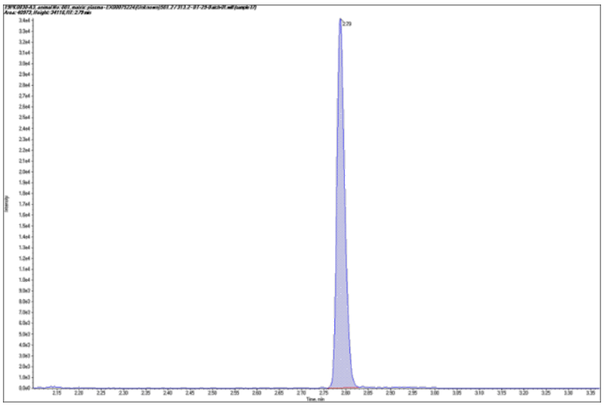

Figure S8. LC-MS chromatogram of an exemplary plasma sample containing FP.

6. Model code

See Supplementary material 2.

7. Calculation of total lung concentrations

Total lung concentrations were calculated based on the concentrations in the individual tissue samples of the upper bronchial tree, the alveolar parenchyma and the remaining lung tissue (see Materials and Methods, 2.4). The concentrations were converted to amounts using the respective sample weights (see Table S4). The total amount in all samples was then divided by the sum of all sample weights. These concentrations were then used for the estimation of PK model parameters. The resulting model predictions can be seen in Figure S9.

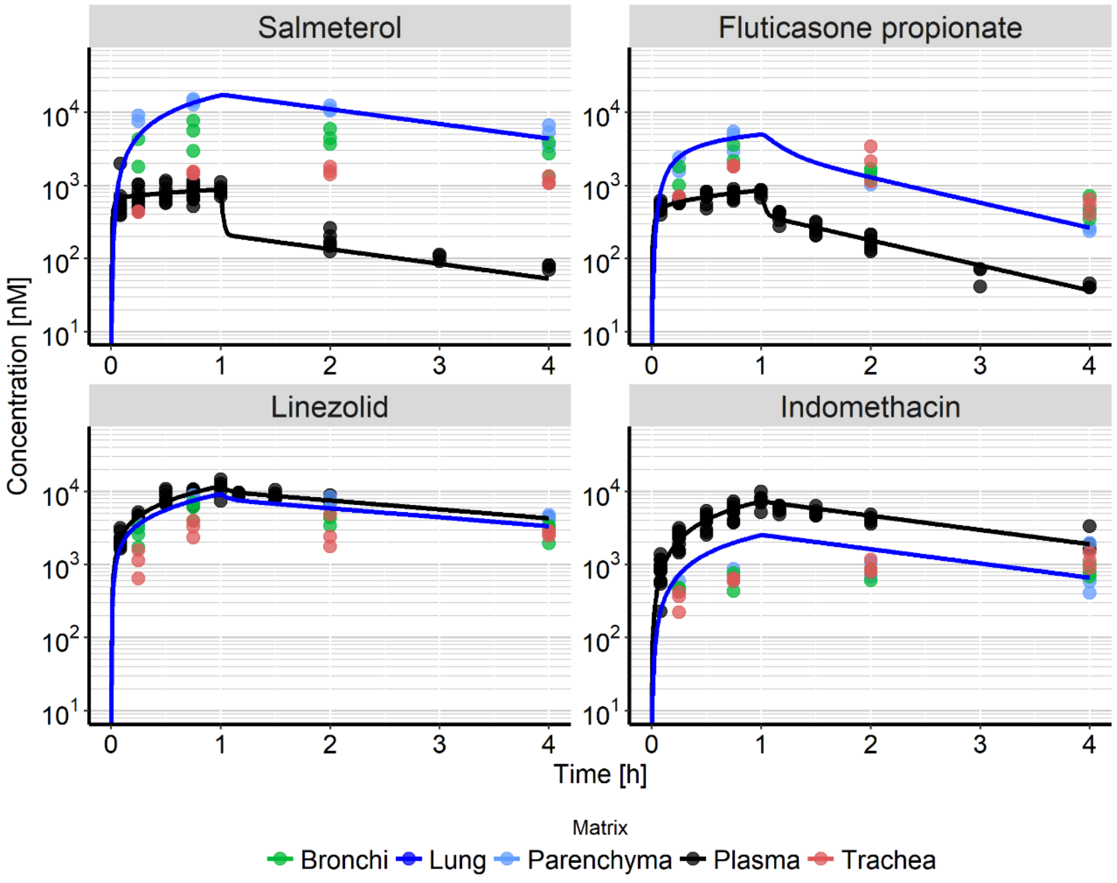

Figure S9. Concentration-time profiles predicted for plasma (black) and total lung in comparison to the observed concentrations in the separate lung tissues.

References:

1. Inagi, T.; MURAMATSU, T.; NAGAI, H.; TERADA, H. Mechanism of indomethacin partition between n-octanol and water. *Chemical and Pharmaceutical Bulletin* **1981**, *29*, 2330-2337.
2. Taylor, R.; Sunderland, B.; Luna, G.; Czarniak, P. Evaluation of the stability of linezolid in aqueous solution and commonly used intravenous fluids. *Drug design, development and therapy* **2017**, *11*, 2087.
3. Chiang, P.-C.; Hu, Y. Simultaneous determination of logd, logp, and pka of drugs by using a reverse phase hplc coupled with a 96-well plate auto injector. *Combinatorial chemistry & high throughput screening* **2009**, *12*, 250-257.
4. Budavari, S.; O'Neil, M.J.; Smith, A.; Heckelman, P.E. *The merck index*. Merck Rahway, NJ: 1989; Vol. 11.
5. Cui, Y.; Lotz, R.; Rapp, H.; Klinder, K.; Himstedt, A.; Sauer, A. Muscle to brain partitioning as measure of transporter-mediated efflux at the rat blood–brain barrier and its implementation into compound optimization in drug discovery. *Pharmaceutics* **2019**, *11*, 595.
