# Supplementary material for: Towards a Quantitative Mechanistic Understanding of Localized Pulmonary Tissue Retention—A Combined In Vivo/In Silico Approach Based on Four Model Drugs
Source: Pharmaceutics. 2020 Apr 29;12(5):408. doi: 10.3390/pharmaceutics12050408 (PMC7284631; doi:10.3390/pharmaceutics12050408)
Supplement: Supplementary file 1 [file pharmaceutics-12-00408-s001.zip › Supplementary_material_2.pdf]

```
test(){
        deriv(A1 = - Cl * C - Cl2 * (C - C2))
        deriv(A2 = Cl2 * (C - C2))

        deriv(Parenchyma = QP * (C - 1/KPP*CP))
        deriv(Bronchi    = QB * (C - 1/KPB*CB))
        deriv(Trachea    = QT * (C - 1/KPT*CT))

        dosepoint(A1)

        C = A1 / V
        C2 = A2 / V2

        Parenchyma_weight = 0.004     # kg parenchyme /kg bodyweight
(assumption: 80% of total lung)
        Bronchi_weight    = 0.0008    # kg parenchyme /kg bodyweight
(assumption: 20% of total lung - Trachea based on sample)
        Trachea_weight    = 0.0002    # Based on sample in the respective
studies

        CP = Parenchyma / Parenchyma_weight # concentration in the alveolar
parenchyma
        CB = Bronchi / Bronchi_weight       # concentration in the bronchi
        CT = Trachea / Trachea_weight       # concentration in the trachea

        error(CEps = 0.1)                   # residual variability plasma
        error(CEps_t = 0.1)                 # residual variability tissue

        observe(CObs = C * (1 + CEps))

        ## Error model
        observe(CObs_P = CP * (1 + CEps_t))
        observe(CObs_B = CB * (1 + CEps_t))
        observe(CObs_T = CT * (1 + CEps_t))

        ## Define covariate -> Compound;  1=FP, 2=Linezolid, 3=Indomethacin,
4=Salmeterol
        fcovariate(CPD())

        ## Define sytemic PK parameters
        stparm(V   = tvVfp*(CPD==1)   + tvVlin*(CPD==2)   + tvVind*(CPD==3)   +
tvVsal*(CPD==4))
        stparm(V2  = tvV2fp*(CPD==1)  + tvV2lin*(CPD==2)  + tvV2ind*(CPD==3)  +
tvV2sal*(CPD==4))
        stparm(Cl  = tvClfp*(CPD==1)  + tvCllin*(CPD==2)  + tvClind*(CPD==3)  +
tvClsal*(CPD==4))
        stparm(Cl2 = tvCl2fp*(CPD==1) + tvCl2lin*(CPD==2) + tvCl2ind*(CPD==3) +
tvCl2sal*(CPD==4))

        ## Define the lung partition coefficients
        stparm(KPP = tvKPPfp*(CPD==1) + tvKPPlin*(CPD==2) + tvKPPind*(CPD==3) +
tvKPPsal*(CPD==4)) # Kp of the alveolar parenchyma
        stparm(KPB = tvKPBfp*(CPD==1) + tvKPBlin*(CPD==2) + tvKPBind*(CPD==3) +
tvKPBsal*(CPD==4)) # Kp of the bronchi
```

```
        stparm(KPT = tvKPTfp*(CPD==1) + tvKPTlin*(CPD==2) + tvKPTind*(CPD==3) +
tvKPTsal*(CPD==4)) # Kp of the trachea

        ## Define the perfusion rates
        stparm(QP = tvQP * (Ff_fp*(CPD==1) + Ff_lin*(CPD==2) + Ff_ind*(CPD==3) +
Ff_sal*(CPD==4))) # alveolar blood flow
        stparm(QB = tvQB * (Ff_fp*(CPD==1) + Ff_lin*(CPD==2) + Ff_ind*(CPD==3) +
Ff_sal*(CPD==4))) # bronchial blood flow
        stparm(QT = tvQT * (Ff_fp*(CPD==1) + Ff_lin*(CPD==2) + Ff_ind*(CPD==3) +
Ff_sal*(CPD==4))) # tracheal blood flow

        fixef(tvQP = c(0, 21, ))
        fixef(tvQB = c(0, 2, ))
        fixef(tvQT = c(0, 0.5, ))

## Initial compound-specific values
# fluticasone propionate
        Ff_fp  = 0.0153    # fraction unbound in plasma

        fixef(tvKPPfp  = c(0, 6, ))
        fixef(tvKPBfp  = c(0, 6, ))
        fixef(tvKPTfp  = c(0, 6, ))

        fixef(tvVfp(freeze)   = c(0, 0.223336, ))
        fixef(tvV2fp(freeze)  = c(0, 2.40999, ))
        fixef(tvClfp(freeze)  = c(0, 3.37369, ))
        fixef(tvCl2fp(freeze) = c(0, 4.71991, ))

# linezolid
        Ff_lin = 0.732    # fraction unbound in plasma

        fixef(tvKPPlin = c(0, 0.9, ))
        fixef(tvKPBlin = c(0, 0.6, ))
        fixef(tvKPTlin = c(0, 0.5, ))

        fixef(tvVlin(freeze)   = c(0, 0.32044, ))
        fixef(tvV2lin(freeze)  = c(0, 0.627917, ))
        fixef(tvCllin(freeze)  = c(0, 0.279411, ))
        fixef(tvCl2lin(freeze) = c(0, 2.79208, ))

# indomethacin
        Ff_ind = 0.024    # fraction unbound in plasma

        fixef(tvKPPind = c(0, 0.3, ))
        fixef(tvKPBind = c(0, 0.3, ))
        fixef(tvKPTind = c(0, 0.3, ))

        fixef(tvVind(freeze)   = c(0, 0.153778, ))
        fixef(tvV2ind(freeze)  = c(0, 1, ))
        fixef(tvClind(freeze)  = c(0, 0.0691472, ))
        fixef(tvCl2ind(freeze) = c(, 0, ))

# salmeterol
        Ff_sal = 0.0139    # fraction unbound in plasma
```

```
        fixef(tvKPPsal = c(0, 45, ))
        fixef(tvKPBsal = c(0, 20, ))
        fixef(tvKPTsal = c(0, 7, ))

        fixef(tvVsal(freeze)   = c(0, 0.12328, ))
        fixef(tvV2sal(freeze)  = c(0, 3.76907, ))
        fixef(tvClsal(freeze)  = c(0, 3.86488, ))
        fixef(tvCl2sal(freeze) = c(0, 3.24267, ))
}
```
