# Supplementary material for: Towards a Quantitative Mechanistic Understanding of Localized Pulmonary Tissue Retention—A Combined In Vivo/In Silico Approach Based on Four Model Drugs
Source: Pharmaceutics. 2020 Apr 29;12(5):408. doi: 10.3390/pharmaceutics12050408 (PMC7284631; doi:10.3390/pharmaceutics12050408)
Supplement: Supplementary file 1 [file pharmaceutics-12-00408-s001.zip › Supplementary_material_TableS4.pdf]

Table S4. Study design and sample weights.

		Bodyweight (BW)				Infusion		Sampling time points (min)		Mean sample weights (g)									
**Drug**	**Study**	**mean (g)**	**%CV**	**N**	**Dose (mg/kg/h)**	**duration (min)**	**rate (mL/kg/h)**	**tissue (min)**	**plasma (min)**	**lung (g)**	**%CV**	**trachea (g)**	**%CV**	**bronchi (g)**	**%CV**	**parenchyma (g)**	**%CV**	**rest lung (g)**	**%CV**
**Salmeterol**[1]																			
	1A	252	2.6	2	2	15	10	15	5, 15	1.13	5.05	0.0477	15.5	0.133	7.99	0.0493	21.6	0.590	16.4
	1B	264	3.0	3	2	45	10	45	'' , 30, 45	1.17	0.70	0.0420	7.01	0.130	12.0	0.0527	29.7	0.627	2.11
	1C	257	1.8	3	2	60	10	120	'', 60, 120	1.18	3.44	0.0533	0.884	0.151	8.19	0.0523	23.7	0.633	8.93
	1D	251	2.6	3	2	60	10	240	'', 240	1.14	4.96	0.0490	18.6	0.127	16.0	0.0530	38.3	0.648	10.2
**Fluticasone Propionate**[1]																			
	2A	321	3.0	2	2	15	10	15	5, 15	1.50	0.739	0.0647	19.1	0.167	25.79	0.109	39.6	0.744	14.1
	2B	329	6.7	3	2	45	10	45	'' , 30, 45	1.53	9.87	0.0687	5.36	0.194	9.67	0.131	14.3	0.604	30.5
	2C	332	5.6	3	2	60	10	120	'', 60, 70, 90, 120	1.48	1.80	0.0703	8.15	0.159	9.80	0.0717	21.7	0.759	11.4
	2D	297	2.7	3	2	60	10	240	'', 180, 240	1.32	2.07	0.0660	21.4	0.125	67.9	0.186	45.7	0.492	31.9
**Linezolid**																			
	3A	280	3.9	3	4	15	10	15	5, 15	1.20	2.97	0.0540	12.1	0.123	19.5	0.0730	32.8	0.640	10.2
	3B	280	1.9	3	4	45	10	45	'' , 30, 45	1.33	1.50	0.0470	4.60	0.139	33.9	0.101	46.7	0.637	10.2
	3C	280	1.2	3	4	60	10	120	'', 60, 70, 90, 120	1.25	9.00	0.0400	7.36	0.143	34.7	0.0640	77.6	0.598	14.0
	3D	279	2.5	3	4	60	10	240	'', 240	1.30	10.4	0.0483	32.7	0.132	27.3	0.104	34.7	0.606	26.6
**Indomethacin**																			
	4A	250	1.1	3	0.5	15	10	15	5, 15	1.10	0.197	0.0423	34.6	0.0960	11.4	0.0387	11.6	0.495	14.1
	4B	277	1.8	3	0.5	45	10	45	'' , 30, 45	1.36	6.50	0.0480	2.95	0.150	11.9	0.119	55.8	0.720	8.60
	4C	290	3.7	3	0.5	60	10	120	'', 60, 70, 90, 120	1.30	10.1	0.0443	26.1	0.149	3.56	0.0600	40.3	0.786	17.7
	4D	259	5.2	3	0.5	60	10	240	'', 240	1.30	18.0	0.0413	8.91	0.118	18.5	0.112	46.7	0.660	12.0
**Total**	**mean**	281								1.29		0.0517		0.140		0.0860		0.640	
	**%CV**	9.3								12.5		25.5		20.6		63.9		20.4	

[1]For both salmeterol and fluticasone propionate, results from one rat designated for tissue sampling after 0.25 h had to be excluded due to para-venous administration.
